# 5-(Indol-2-yl)pyrazolo[3,4-*b*]pyridines as a New Family of TASK-3 Channel Blockers: A Pharmacophore-Based Regioselective Synthesis

**DOI:** 10.3390/molecules26133897

**Published:** 2021-06-25

**Authors:** David Ramírez, Melissa Mejia-Gutierrez, Braulio Insuasty, Susanne Rinné, Aytug K. Kiper, Magdalena Platzk, Thomas Müller, Niels Decher, Jairo Quiroga, Pedro De-la-Torre, Wendy González

**Affiliations:** 1 Instituto de Ciencias Biomédicas, Universidad Autónoma de Chile, Llano Subercaseaux 2801-Piso 5, Santiago 8900000, Chile; 2 Heterocyclic Compounds Research Group, Department of Chemistry, Universidad del Valle, A.A, Cali 760031, Colombia; melissa.mejia@correounivalle.edu.co (M.M.-G.); braulio.insuasty@correounivalle.edu.co (B.I.); jairo.quiroga@correounivalle.edu.co (J.Q.); 3 Institute for Physiology and Pathophysiology, Vegetative Physiology and Center for Mind, Brain and Behavior (CMBB), Philipps-University of Marburg, Deutschhausstraße 2, 35037 Marburg, Germany; rinne@staff.uni-marburg.de (S.R.); aytug.kiper@staff.uni-marburg.de (A.K.K.); decher@staff.uni-marburg.de (N.D.); 4 Joint Pulmonary Drug Discovery Lab Bayer-MGH, Boston, MA 02114, USA; magdalena.platzk@bayer.com; 5 Bayer AG, Research & Development, Pharmaceuticals, D-42096 Wuppertal, Germany; thomas.mueller6@bayer.com; 6 Department of Otolaryngology, Harvard Medical School and Massachusetts Eye and Ear, 243 Charles St, Boston, MA 02114, USA; 7 Caribe Therapeutics, Vía 40 No. 69-111, Oficina 804 A, Barranquilla 080002, Colombia; 8 Centro de Bioinformática, Simulación y Modelado (CBSM), Facultad de Ingeniería, Universidad de Talca, Poniente No. 1141, Talca 3460000, Chile; 9 Millennium Nucleus of Ion Channels-Associated Diseases (MiNICAD), Universidad de Talca, Talca 3460000, Chile

**Keywords:** TASK-3 channel blockers, pyrazolo[3,4-*b*]pyridines, molecular docking, drug design, pharmacophore

## Abstract

TASK channels belong to the two-pore-domain potassium (K_2P_) channels subfamily. These channels modulate cellular excitability, input resistance, and response to synaptic stimulation. TASK-channel inhibition led to membrane depolarization. TASK-3 is expressed in different cancer cell types and neurons. Thus, the discovery of novel TASK-3 inhibitors makes these bioactive compounds very appealing to explore new cancer and neurological therapies. TASK-3 channel blockers are very limited to date, and only a few heterofused compounds have been reported in the literature. In this article, we combined a pharmacophore hypothesis with molecular docking to address for the first time the rational design, synthesis, and evaluation of 5-(indol-2-yl)pyrazolo[3,4-*b*]pyridines as a novel family of human TASK-3 channel blockers. Representative compounds of the synthesized library were assessed against TASK-3 using Fluorometric imaging plate reader—Membrane Potential assay (FMP). Inhibitory properties were validated using two-electrode voltage-clamp (TEVC) methods. We identified one active hit compound (**MM-3b**) with our systematic pipeline, exhibiting an IC_50_ ≈ 30 μM. Molecular docking models suggest that compound **MM-3b** binds to TASK-3 at the bottom of the selectivity filter in the central cavity, similar to other described TASK-3 blockers such as A1899 and PK-THPP. Our in silico and experimental studies provide a new tool to predict and design novel TASK-3 channel blockers.

## 1. Introduction

Leak or background two-pore domain potassium (K_2P_) channels are a superfamily of proteins that selectively modulate the flow of potassium (K^+^) ions across the plasma membrane of living cells. These membrane proteins are expressed in the central and peripheral nervous systems (playing a critical role in regulating membrane potential and input resistance in neurons) [1], astrocytes (mediating fast and slow glutamate release) [2], the cardiovascular system (upregulation of atrial K_2P_ currents was described in patients suffering from atrial fibrillation) [3], and the cochlea (likely regulating endolymphatic K^+^ homeostasis in the inner ear) [4]. Their expression is up- and downregulated in different cancer cells [5], suggesting a wide spectrum of functions to these channels [6], including, but not limited to cell signaling, behavior [1], normal hearing [4], breathing control [7], anesthesia [8], and tumorigenesis [5]. These aspects provide unique attributes to this superfamily of proteins as potential druggable targets for drug discovery [6].

TASK-1 (also known as *KCNK3* and K_2P_3.1), TASK-3 (*KCNK9* and K_2P_9.1), and TASK-5 (*KCNK15* and K_2P_15.1) are members of the TASK (tandem of pore domains in a weak inwardly rectifying K^+^ channel [TWIK]-related acid-sensitive K^+^) channel subfamily of proteins [6], which share between 51 and 59% of protein-sequence identity [9]. Protein sequence analyses and homology modeling of TASK-1, -3, -5, and the recently solved TASK-1 crystallographic structures (PDB codes: 6RV2, 6RV3, and 6RV4 [10]) show that these TASK channels share similar folding and topology with other K_2P_ channels [6].

K_2P_ channels have two pore domains and four transmembrane domains (2P/4TM) assembled as homotypic [11] and heterotypic dimers. Moreover, two extracellular ion pathways (EIP) formed by two large extracellular linkers from the M1 to the first pore loop (M1-P1 linker), a selectivity filter at the central cavity, and two dynamic ligand-binding open cavity sites facing the cell membrane (known as side fenestrations) are typical structural features of these biomolecules [12]. TASK channels present a lower gate (X-gate) created by the interaction of the two crossed C-terminal M4 transmembrane helices at the vestibule entrance. These features provide unique gating mechanisms and electrophysiological properties to these TASK channels [10]. All these functional characteristics of TASK channels could be modulated by their capacity to selectively bind inhibitors [6].

**Figure 1:**
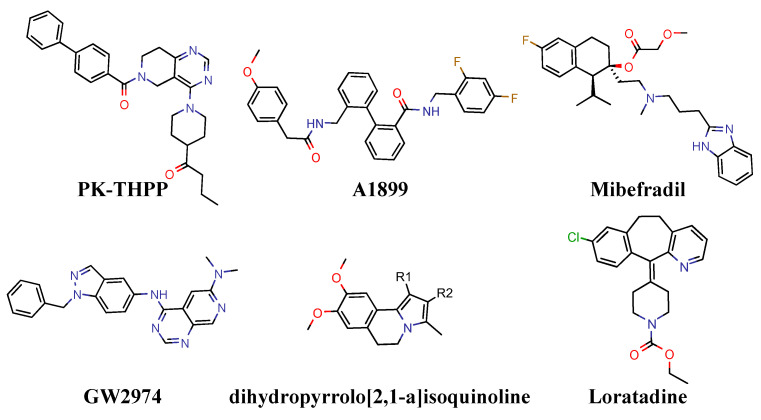
Representative compounds reported as TASK-3 blockers [6].

Despite the druggable capacity of TASK-3, the exploration of the chemical space of potential new TASK-3 modulators has been poorly studied [13]. A very limited variety of heterocyclic compounds such as dihydropyrrolo[2,1-*a*]isoquinoline [14], tetrahydropyrido[4,3-*d*]pyrimidine (PK-THPP and derivatives [13]), pyridine (loratadine), benzopyrazole (GW2974), and benzimidazole (mibefradil) derivatives [15] have been reported as TASK-3 blockers, as well as biphenyl blockers such as A1899 [6], suggesting that a rational molecular hybridization of these heterocyclic moieties in a single scaffold should be explored to discover more potent TASK-3 inhibitors (Figure 1).

The benzopyrazole derivative GW2974 [15] shares a similar scaffold to pyrazolo[3,4-*b*]pyridine compounds, which we have extensively studied in our labs [16,17,18], including their analogs [19]. Interestingly, when these moieties are rationally assembled in a single scaffold, such as pyrazolo[3,4-*b*]pyridines, they have shown antitumoral [20], antibacterial/microbial [21], trypanocidal [22], antidepressant, anxiolytic [23], antiviral [24], and anti-inflammatory [25] activities, making these structural moieties an invaluable source of new bioactive molecules of interest in the medicinal chemistry field.

In this study, we report a three-step systematic pipeline where a new library of pyrazolo[3,4-*b*]pyridine compounds (**MM-3a–i**) was rationally designed, synthesized, and tested as potential novel human (*h*) TASK-3 channel antagonists. First, the compounds **MM-3a–i** were hypothesized and designed using the TASK-3 blocker pharmacophore previously developed and used to discover other selective TASK-3 blockers [9]; then, we synthesized **MM-3a–i** via cyclocondensation reactions between 2-(3,3-dimethylindolin-2-ylidene)malonaldehyde **1** and a library of 5-aminopyrazoles **2a–i**. The synthesized compounds were fitted into our pharmacophore model to compare their structural similarity and common molecular features (two hydrogen bond acceptors and one aromatic group) present in other TASK-3 channel inhibitors [6,9]. Finally, molecular docking models of the pyrazolo[3,4-*b*]pyridines—TASK-3 complexes were predicted to select the most promising hits for electrophysiological assays.

## 2. Results

### 2.1. Design of Novel TASK-3 Blockers

**Figure 2:**
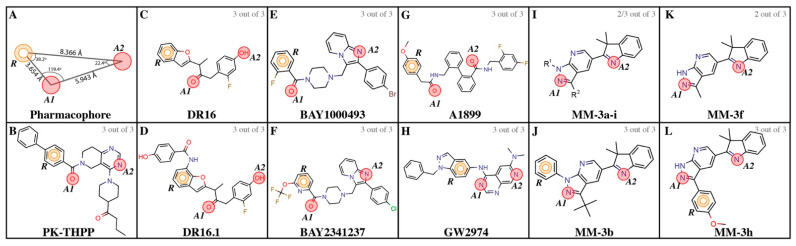
Pharmacophore model and designed compounds. (**A**) Three-point pharmacophore of TASK-3 blockers showing pharmacophoric features *A1* (hydrogen-bond acceptor 1), *A2* (hydrogen-bond acceptor 2) in red, and *R* (aromatic ring) in orange. (**B**–**H**) Compounds PK-THPP, DR16, DR16.1, BAY1000493, BAY2341237, A1899, and GW2974 with reported antagonist activity against TASK-3, respectively. (**I**) Pyrazolo[3,4-*b*]pyridine series, R^1^: H, Ph, 4-Cl-C_6_H_4_, 2-ClC_6_H_4_, 4-NCC_6_H_4_, and 4-BrC_6_H_4_; R^2^: CH_3_, *t*Bu, Ph, 4-CH_3_OC_6_H_4_, 4-BrC_6_H_4_, and 4-CH_3_C_6_H_4_. (**J**–**L)** Compounds **MM-3b**, **MM-3f**, and **MM-3h** designed in the present work, respectively. **MM-3b** and **MM-3h** are shown with the three-point pharmacophore *A1, A2,* and the required *R* pharmacophoric feature; **MM-3f** lacks this *R*-aromatic feature.

Recently, our group modeled a three-point common pharmacophore from some known TASK-3 blockers (Figure 2A) and reported two new TASK-3 antagonists (DR16 and DR16.1) with potency in the micromolar range [9] (Figure 2C,D). This pharmacophore model presents one aromatic ring (*R*) and two hydrogen bond acceptors (*A1* and *A2*), which are conserved in PK-THPP (IC_50_ = 35 nM), A1899 (IC_50_ = 320 nM), BAY1000493 (EC_50_ = 15.1 nM), and BAY2341237 (EC_50_ = 2.3 nM), which are some of the most potent TASK blockers reported to date [10,13] (Figure 2B,E–G) (IC_50_: molar concentration of an inhibitory agonist that reduces a response by 50% of the maximal inhibition to be attained; EC_50_: molar concentration of an agonist producing 50% of the maximal possible effect of that agonist. The action of the agonist may be stimulatory or inhibitory [26]). As a starting point, we used as a reference these pharmacophoric features previously described and the structural similarity between the TASK-3 blocker GW2974 benzopyrazole [15] and pyrazolo [3,4-*b*]pyridine compounds, which has been one of the most well studied scaffolds in our laboratory in the last years [27] (Figure 2H). Our pharmacophore-based preliminary design (upon reagent availability) resulted in nine compound candidates (Figure 2I–L). Here, the pyrazolo[3,4-*b*]pyridine scaffold is sketched with the same two hydrogen bond acceptors *A1* and *A2* in red circles (Figure 2I); an aromatic ring *R* (orange circle) as a third site, also present in GW2974 (Figure 2H), located at the N1 or the C3 atoms of the pyrazole moiety (compounds **MM-3b** and **MM-3h**, respectively. Figure 2J,L), or it is removed from the pyrazolo[3,4-*b*]pyridine scaffold (compound **MM-3f**, Figure 2K). Our rational design allowed us to explore the influence of *R* on the activity of the **MM-3i–a** series.

### 2.2. Synthesis of Designed TASK-3 Blockers

**Scheme 1:**
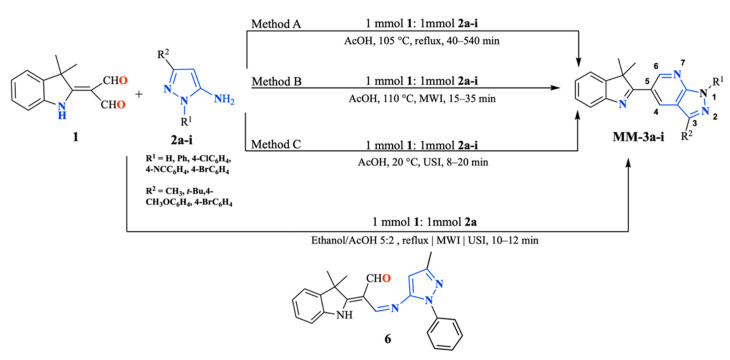
Synthesis of new 5-(indol-2-yl)pyrazolo[3,4-*b*]pyridines **MM-3a–i**.

To approach the pharmacophore-based regioselective synthesis of the 5-(indol-2-yl)pyrazolo[3,4-*b*]pyridines **MM-3a–i** of interest, we synthesized the 2-(3,3-dimethylindolin-2-ylidene)malonaldehyde **1** [28,29] and a library of 5-aminopyrazoles **2a–i** as building blocks (see Section 4.1). The cyclocondensation reaction between the dialdehyde **1** and 5-aminopyrazole **2a** did not progress under reflux conditions using ethanol as solvent or basic conditions using triethylamine, as reported for similar cyclocondensation reactions [30]. Then, we explored three additional synthetic protocols under acidic conditions: method A: using acetic acid (AcOH) as solvent under conventional heating/reflux, method B: AcOH under microwave irradiation (MWI) at 110 °C and 200 Watts (W), and method C: AcOH assisted by ultrasound irradiation (USI) at 20 KHz/500 W at 20 °C (room temperature, rt) to ascertain the most useful methodology and regioselectively obtain the designed compounds **MM-3**. Method C provided the best results with the shortest reaction times (8–20 min), highest yields (65–90%), and purity. The progress of the reactions was verified using thin layer chromatography (TLC), evidencing the formation of a single product for each reaction, which was isolated by simple filtration (Scheme 1 and Table 1). The possible formation of different regioisomers in the filtrated solids were discarded by nuclear magnetic resonance (NMR) experiments and mass spectrometry (see Scheme 1, Materials and Methods section, Supplementary Methods section, Figures S2–S22, and Supplementary Scheme S1).

**Scheme 2:**
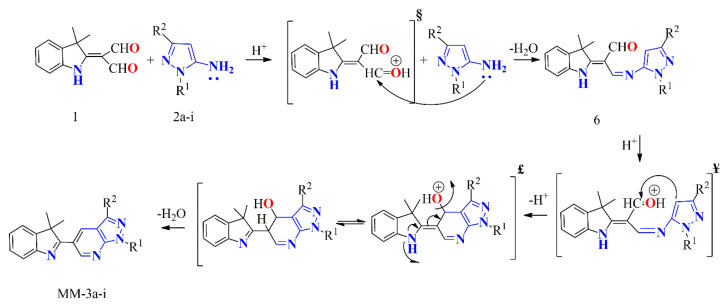
Proposed mechanistic route to obtain **MM-3a–i** via intermediate **6**. §: Intermediate of dialdehyde **1** with a protonated formyl group. ¥: Intermediate of compound **6** (R^2^: Ph) with a protonated formyl group. £: Cyclocondensed intermediate of compound **6**.

In all cases, the regioisomers **MM-3a–i** were selectively obtained as unique products, and their structures were confirmed by ^1^H and ^13^C NMR (mono (1D) and two-dimensional (2D)) and mass spectrometry (see Section 4.1). Particularly, the ^1^H NMR analysis confirms obtaining the desired compounds. NMR spectra show two new singlets (for **MM-3a**, **3e**, **3h**) or doublets (for **MM-3b**, **3c**, **3d**, **3f**, **3g**, and **3i**) with *J* coupling of 1–2.1 Hz corresponding to the new H-6 (≈9.0 ppm) and H-4 (≈8.0 ppm) protons of the pyridine moiety (see atom numbering in Scheme 1 and Table 1). The absence of the proton corresponding to the formyl group of compounds **4** discards these obtained derivatives (Scheme S1). In addition, the absence of the H-4 pyrazole protons of the precursors **2** precludes the presence of compound **5** (Scheme S1) and is consistent with the pharmacophore-base designed regioselective products **MM-3a–i** (Scheme 1). Compounds **MM-3f**, **3g**, **3h**, and **3i** display one singlet in the region of 13.0–13.5 ppm corresponding to the NH of the free pyrazole of the pyrazolo[3,4-*b*]pyridines. To further study the possible synthetic route for the formation of the compounds **MM-3a–i**, we mixed equimolar amounts of the dialdehyde **1** and the 5-aminopyrazole **2a** in an ethanol/AcOH 5:2 ratio. This mixture was subjected to USI, MWI, and reflux for 10–12 min. Interestingly, with all these three methods, a pure and stable imine (intermediate **6**) was isolated and characterized (Scheme 1. See also Table 1, Scheme 2, and Methods section). This outcome allowed us to propose a possible mechanistic route for obtaining the compounds **MM-3a–i** via a rapid two-step cyclocondensation reaction. Briefly: first, the condensation reaction between the amine group from the 5-aminopyrazole **2a** and a protonated formyl group from **1** produced the intermediate imine **6**, and second, the addition of the C-4 pyrazole moiety to the remaining protonated formyl group (intermediate ¥) led to compounds **MM-3a** (Scheme 2).

The 2-(3,3-dimethylindolin-2-ylidene)malonaldehyde **1** contains three electrophilic sites, including the two formyl groups and the α,β-unsaturated system, which are susceptible to a nucleophilic attack from the aminopyrazole moiety [28,29] (Scheme 1 and Scheme 2). Due to these electrophilic sites, it was reasonable to think about three potential products **MM-3a–i**, **4**, and/or **5**, as represented in Scheme 1 and Scheme S1. A Michael-reaction type to the α,β-unsaturated system of **1** could provide the products **4** harboring a free formyl group, while a dual nucleophilic addition to both carbonyl groups of **1** could provide the products **5** (Scheme S1). Nevertheless, method C promoted the synthesis of the new compounds **MM-3a–i** via a regioselective nucleophilic attack of the pyrazole C-4 carbon atom to the second available protonated formyl group from intermediate **6** (¥). Overall, the USI was the most effective method, and it surpassed methods A and B in terms of decreasing the reaction times while increasing yields parallel to obtaining regioselectively the 5-(indol-2-yl)pyrazolo[3,4-*b*]pyridines **MM-3a–i**. These results could be explained by the physical phenomenon of acoustic cavitation, which has extensively provided more benefits in a variety of synthetic reactions [30,32]. Regardless of the synthetic method, we do not see a clear influence of the substitution pattern of the pyrazole ring on the obtained times and reaction yields (Table 1).

### 2.3. Molecular Docking and ADME/Tox Predictions

We modeled the nine designed and synthesized **MM-3** compounds (and intermediate **6**, Table 1) interacting with *h*TASK-3 channels to optimize synthetic and biological resources (compounds were synthetized upon reagent availability) and minimize time. The pyrazolo[3,4-*b*]pyridines derivatives **MM-3a–i** and the intermediate **6** were docked into the central cavity of *h*TASK-3, which was modeled using the *h*TASK-1 crystallographic structure (PDB code: 6RV2) as a template [10]. TASK-1 and TASK-3 share 59% of sequence identity [33]. There is small difference between the central cavities of TASK-1 and TASK-3 channels. In TASK-1 the residue M247 is replaced to L247 [34], reason why the selected crystallographic structure is a suitable template for generating an optimal *h*TASK-3 homology model. For membrane proteins, sequence identities with ≥30% provide homology models with RMSDs < 2 Å relative to the native structures in the TM regions [35]. To assess our docking simulations, we included the known TASK-3 blockers A1899 [34,36] and PK-THPP [13] (which share the same three-point pharmacophore model, Figure 2) as positive controls, whose binding site in TASK channels is known to be in the central cavity. Docking results are presented in Table 2.

The known TASK-3 blockers A1899 and PK-THPP showed the best docking scores (−9.478 kcal/mol and −8.961 kcal/mol, respectively), which are in agreement with their reported inhibitory potency against TASK-3 channels (A1899 IC_50_ = 0.32 µM [34] and PK-THPP IC_50_ = 35 nM [13]), followed by the pyrazolo[3,4-*b*]pyridines **MM-3b** (−8.485 kcal/mol) and **MM-3h** (−8.124 kcal/mol).

We also studied the influence of the structural features of the pyrazolo[3,4-*b*]pyridines **MM-3a–i** on their potential biological properties using physicochemical descriptors, which were calculated for the reported blockers A1899, PK-THPP, and compounds **MM-3a–i**, as well as intermediate **6**. Predictions included the molecular weight (MW), number of rotatable bonds, total number of hydrogen bond acceptors (HB-A) and donors (HB-D), and topological polar surface area (TPSA). Furthermore, the lipophilicity, water solubility, and pharmacokinetic properties were calculated (Table 3 and Table S1). Compounds reported in this work have a MW < 500 g/mol, which is optimal for a potential lead drug. All calculated physicochemical descriptors and pharmacokinetics properties are in the defined acceptable ranges to assess drug-likeness according to the Lipinski’s rule of five, with some exceptions regarding lipophilicity (Log P_o/w_) [37].

We used the calculated properties to predict drug-likeness according to the Ghose, Veber, Egan, and Muegge rules [38] (Table S1), and we found that compounds **MM-3a**, **3d**, **3f**, **3g**, and **3h** did not present any rule violation. Even though **MM-3b** presented three violations, these cannot be compared to those presented by A1899, with a total of eight. **MM-3b** violations are related to its high Log P_o/w_ = 5.42, which can be compared to A1899 (Log P_o/w_ = 5.34). The other predicted physicochemical descriptors and pharmacokinetics properties of **MM-3b** are within the suggested values [38]. This in silico analysis of the predicted physicochemical descriptors for compounds **MM-3a–i** (and intermediate **6**) suggests a positive contribution of substituents of the obtained pyrazolo[3,4-*b*]pyridines for their plausible oral use. Further experiments are required to validate these results.

In the tridimensional space, our pharmacophore model suggests that the designed compounds should have the aromatic group (*R*) located at distances of 3.654 Å and 8.366 Å from *A1* and *A2*, respectively, forming an angle of 119.4 Å respect to *A1*, as a common requirement for designing any potential *h*TASK-3 blocker [9] (Figure 2A). To choose the compounds from our set to be used for biological assays, we considered different criteria and hypothesized that an active TASK-3 blocker should fit with the three-point pharmacophore as a minimum requirement. Additionally, we consider that the *R* feature is key for establishing TASK-3–ligand blocking interactions, since the only two co-crystallized TASK blockers (PDBs: 6RV3-BAY1000493 and 6RV4-BAY2341237) mostly present hydrophobic interactions at the pore region. When this pharmacophore is altered by eliminating the *R* group, we expect no biological activity, while varying the *R* group at N1 and C3 sites, the compounds might be active. To validate this hypothesis, we mainly relied on our validated three-point pharmacophore model. Therefore, among the compounds that fully comply with this model (**MM-3a-e**, Table 1), we chose **MM-3b**, since this is the one with the best docking score (−8.485 kcal/mol, Table 2). Furthermore, **MM-3b** is predicted to mediate similar interactions with TASK-3 residues T93, I118, L122, F125, Q126, T199, L232, I235, L239, L244, and L247, compared to the TASK-1 crystal structure interacting with BAY2341237 (PDB: 6R4V, Figure 2, and Figure S23).

In addition, we chose **MM-3f** for the biological assessment to study the effect when the *R* group is removed because it fit two out of the three pharmacophore features (Figure 2I) and also because it showed lower docking scores (−7.409 kcal/mol, Table 2) among compounds without the *R* substituent (**MM-3f** and **3g**). The *tert*-butyl group at the C3 carbon atom of **MM-3b** was replaced with a methyl group in **MM-3f**, which is a less steric substituent. Finally, to study the effect the *R* group had when it occupies the C3 position on the pyrazole ring, the options were **MM-3h** and **MM-3i**; between both compounds, we chose **MM-3h**, since it had the lower docking score (−8.124 kcal/mol, Table 2). This could lead to a better understanding of the pharmacophore–bioactivity relationship and the contribution of the substitution pattern among compounds **MM-3b**, **3f**, and **3h**.

### 2.4. Biological Assays of Designed Compounds

**Figure 3:**
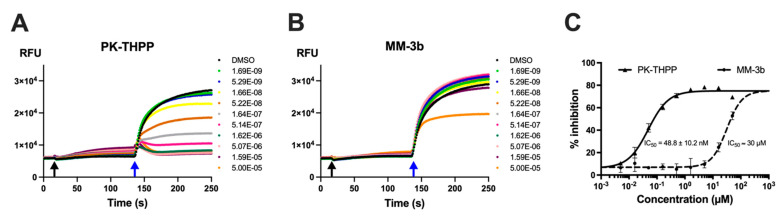
Changes in fluorescence intensities (RFU—Relative Fluorescence Units) of the FMP dye in FLIPR in response to increasing compound PK-THPP (**A**) and **MM-3b** (**B**) concentration (1.69 × 10^−9^ to 5.00 x 10^−5^ M). Black and blue arrows indicate the time where the compounds in different concentrations (M) and KCl (40 mM) were added, respectively. (**C**) Dose–response curves of fluorescence changes evoked by the TASK-3 channels blockers **MM-3b** and PK-THPP. Least-squares regression model (four-parameter logistic regression) was employed for curve fitting using GraphPad Prism version 9.0.0 (GraphPad Software, San Diego, CA, USA). We found that the maximal percentage of inhibition for control compound (PK-THPP) was 75%, so we applied a 75% constrain to the top of the **MM-3b** curve fitting. The data shown are mean values from quadruplicate wells.

The three pyrazolo[3,4-*b*]pyridines analogs **MM-3b**, **MM-3f**, and **MM-3h**, as well as the reported blocker PK-THPP (used as positive control), were tested against *human* TASK-3 channels using the Fluorometric imaging plate reader (FLIPR)–Membrane Potential assay kit (FMP) [39] (see methods). Only compound **MM-3b** showed inhibitory effects against potassium-induced depolarization by TASK-3 (data for compounds **MM-3f** and **MM-3h** is shown in Figure S1) in a stable recombinant CHO-*h*TASK-3 cell line. Figure 3A shows the ΔFluorescence in TASK-3–transfected CHO-K1 cells in response to depolarization induced by application of 40 mM KCl.

After measuring baseline fluorescence for 10 s, the compounds were added (Figure 3A, black arrow), and the fluorescence signal was detected for 120 s. Then, the external potassium concentration was increased (from 2 to 40 mM) to induce a change in the membrane potential (Figure 3A,B, blue arrow), and the signal was detected for another 120 s. The dose–response curves (Figure 3C) showed the inhibitory activity of compound **MM-3b** against *h*TASK-3 channels (IC_50_ ≈ 30 µM). The control PK-THPP exhibited an IC_50_ = 48.8 ± 10.2 nM.

**Figure 4:**
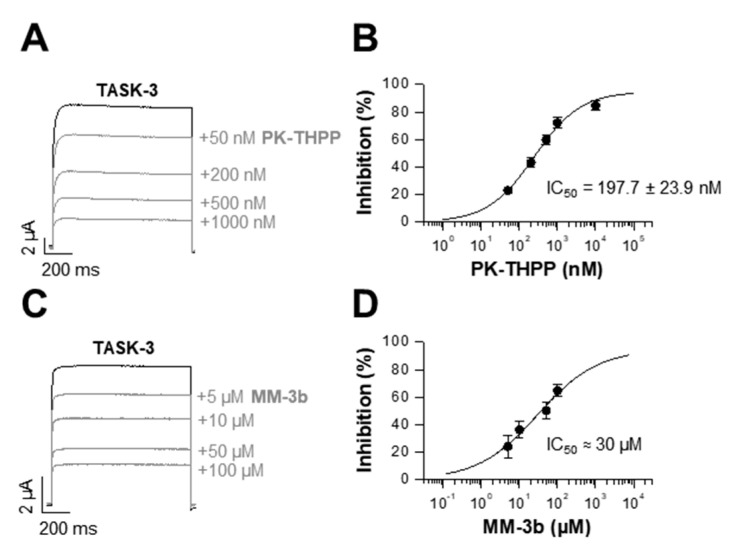
*Human* TASK-3 channels recorded with a voltage-step protocol (1000-ms steps from –80 to +40 mV) before and after application of PK-THPP (**A**) and **MM-3b** (**C**) at different concentrations. Dose–response curves of the *h*TASK-3 channels blockers PK-THPP (**B**) and **MM-3b** (**D**). All data presented as mean ± s.e.m. A least-squares regression model (four-parameter logistic regression) was employed for curve fitting using GraphPad Prism version 9.0.0 (GraphPad Software, San Diego, CA, USA). Due to the limited solubility of **MM-3b** at concentrations higher than 100 µM, the IC_50_ of **MM-3b** was calculated with constrains for a theoretical maximal inhibition at 95%.

To further corroborate our FMP results, we measured the whole cell currents of *h*TASK-3 expressed in *Xenopus* oocytes using two-electrode voltage-clamp (TEVC) recordings, which were analyzed at +40 mV before and after the application of the control compound PK-THPP and the pyrazolo[3,4-*b*]pyridine **MM-3b**, respectively. We corroborated that **MM-3b** is a novel *h*TASK-3 channel blocker with antagonist activity in the low-micromolar range (IC_50_ ≈ 30 µM) (Figure 4).

### 2.5. Proposed Interactions of MM-3b with Residues of the TASK-3 Binding Site

**Figure 5:**
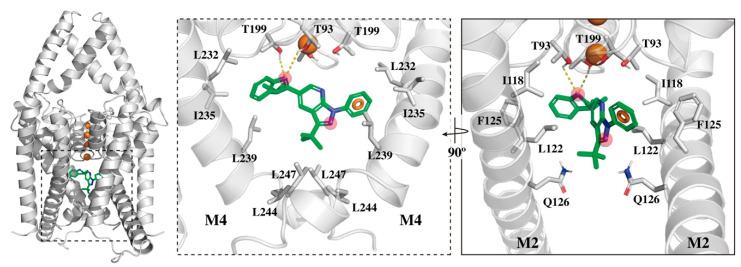
Proposed binding mode of **MM-3b** in TASK-3 central cavity. (Front view) Left, **MM-3b** interactions with TASK-3. (Side view) Right, zoomed binding site with key residues represented as sticks and hydrogen bonds with T93 and T199 residues are shown (dashed lines). The three-point pharmacophore model depicting one aromatic ring (orange circle) and two hydrogen bond acceptors (red spheres) is presented.

Molecular docking models of the **MM-3b**–TASK-3 complexes suggest that this compound is located in the central cavity at the bottom of the selectivity filter of TASK-3, establishing hydrophobic interactions with the following amino acids: I118, L122, F125, Q126, L232, L235, L239, L244, and L247. Hydrogen bonds were also identified between the *A2* (hydrogen-bond acceptor 2—Figure 2) and T93 as well as T199 at the bottom of the selectivity filter (Figure 5). Our results are in line with those reported by our group, since the *R* pharmacophoric feature is oriented toward the M2 and M4 transmembrane segments establishing contacts with I118 and L122, which are two key residues identified in both A1899 [36] and PK-THPP [40] binding sites. Additionally, our results correlate well with crystallographic data for TASK channel blockers (PDB code 6RV3), where the ligand BAY1000493 interacts in the central cavity establishing mainly hydrophobic interactions (Figure S23).

## 3. Discussion

In this study, we used a three-point pharmacophore model of TASK-3 blockers to rationally design novel TASK-3 antagonists. To synthesize the designed molecules **MM-3a–i**, we implemented three different methodologies under acidic conditions (Methods A, B, and C) to compare the effects of these reaction parameters in terms of reaction time, yield, purity, and outcomes. Under Method A, the products **MM-3a–i** were obtained in 33–73% yield with high purity and the longest reaction times (40–540 min). However, using MWI (Method B), the synthesis proceeded in shorter reaction times (15–35 min), but due to the presence of byproducts (not isolated), this method provided the lowest reaction yields (20–40%). When the acidic reaction mixture was subjected to USI as an energy source at room temperature (Method C), the compounds **MM-3a–i** were obtained with the highest yields (70–90%) and shortest reaction times (8–20 min). These outcomes suggest that acidic conditions under USI offer the most suitable strategy to obtain the 5-(indol-2-yl)pyrazolo[3,4-*b*]pyridines **MM-3a–i** compared to the other tested energy sources. In all cases, the regioisomers **MM-3a–i** were selectively obtained as unique products. Singlet or doublet signals for protons H-4 (≈9.0 ppm) and H-6 (≈8.0 ppm) of the new pyridine moiety corroborate our pharmacophore-based regioselective synthesis of compounds **MM-3a–i** as structural analogs of the *h*TASK-3 channel blocker benzopyrazole GW2974 [15] (isomers **4** or **5** were not isolated, Scheme 1, and Scheme S1). Intermediate **6** was isolated when an ethanol/AcOH 5:2 ratio of was used. The acquisition and characterization of **6** allowed us to suggest a possible mechanistic route to obtain the **MM-3a–i** target compounds. First, the protonation of one of the dialdehyde **1** formyl groups would promote the addition of the nucleophilic amino group of the 5-aminopyrazoles **2a–i**. In this step, the imine is produced with subsequent dehydration; then, the protonation of the second formyl group of **1** will contribute to the addition of the second nucleophilic region of **2a–i** with the final dehydration and formation of **MM-3a–i** (Scheme 2).

According to our pharmacophore-based structural design and regioselective synthetic protocol of our hit compounds, we demonstrated that the USI method can be used to easily synthesize larger libraries of derivatives under ultrasound-assisted conditions to expand the chemical space of our pyrazolo[3,4-*b*]pyridine compounds as novel *h*TASK-3 channel blockers.

On the other hand, the designed 5-(indol-2-yl)pyrazolo[3,4-*b*]pyridines **MM-3a–i** and the intermediated **6** were docked into the TASK-3 central cavity, where known blockers such as PK-THPP [40,41] and A1899 [34,36] (sharing the same pharmacophoric features, Figure 2) interact with this protein channel. Compounds **MM-3b** and **MM-3h** presented the best docking scores (Table 2) in comparison to PK-THPP and A1899 (used as positive controls). Despite **MM-3h** showing a docking score of −8.124 Kcal/mol, a change of the *R* feature from the N1 to the C3 position on the pyrazole ring suggests that **MM-3h** does not successfully fit to our pharmacophoric model (compared to **MM-3b**), which is reflected in its lack of activity against TASK-3 channels (Figure S1). This finding allowed us to hypothesize that the presence of the *R* aromatic substituents likely plays a key role in designing any compound as a potential TASK-3 channel blocker. To test our pharmacophore-based hypothesis, we included compound **MM-3f** in our experimental validation, since it lacks this aromatic pharmacophoric feature (Figure 2K). After analyzing the predicted ADME/Tox properties for the compounds reported here, we conclude that all the physicochemical descriptors and pharmacokinetics properties predicted for the compounds **MM-3a–i** (including intermediate **6**) are comparable to acceptable drug-likeness ranges. Interestingly, **MM-3b** presented a Log P_o/w_ = 5.42, which is comparable to the predicted value for the known A1899 TASK-3 blocker (Table 3), suggesting similar lipophilicity between these compounds.

Since it is not easy to choose which representative molecule of a given congeneric series should be scaled up to conduct biological activity assays, in this study, we combine different criteria to select the derivatives of the MM-3 series and assess their TASK-3 channel inhibitory activity. For this reason, we mainly focused on a pharmacophore-based approach and used molecular docking and ADME/tox properties as secondary selection criteria. Three representative compounds were chosen: **MM-3b**, which fulfills all the pharmacophoric features previously validated and described for other TAKS-3 inhibitors (Figure 2), **MM-3f**, which lacks the *R*-aromatic group, a pharmacophoric feature that we consider essential for the interaction with TASK-3 channels, and **MM-3h**, in which the *R* feature was moved from the N1 to C3 position in the pyrazole ring. Based on our pharmacophore design, the compounds **MM-3b**, **MM-3f**, and **MM-3h** were selected for experimental validation using PK-THPP as a positive control. The validation was carried out in two steps: first, we measured the ability to inhibit *h*TASK-3 channels through fluorometric imaging plate reader (FLIPR)–membrane potential assays (FMP), and we found that only **MM-3b** blocks potassium currents in *h*TASK-3 channels with potency in the low micromolar range. Second, we used TEVC experiments and confirmed that **MM-3b** is indeed a novel inhibitor of *h*TASK-3 channels (IC_50_ ≈ 30 µM).

## 4. Materials and Methods

### 4.1. Chemistry

Three methods (A, B, and C) were used to obtain compounds of interest **MM-3a–i**, and the progress of the reactions was monitored by thin-layer chromatography (TLC) with visualization of spots by UV light. TLC analyses were performed on Merck TLC-plates aluminum silica gel 60 F_254._ All the reagents and solvents (analytical grade) were commercially available and used without any further purification. Ultrasonic experiments were carried out on a focused ultrasonic reactor (500 W/20 KHz Fisher Scientific). Microwave experiments were carried out on a focused microwave reactor (300 W CEM Discover). Melting points were determined in a Buchi Melting Point Apparatus and are uncorrected. IR spectra, at 500–400 cm^−1^, were recorded on a Shimadzu 8400 FT-IR spectrophotometer, this equipment is located at the Universidad del Valle, Cali, Colombia, and it was manufactured by Shimadzu Corporation, Kyoto, Japan. The ^1^H- and ^13^C NMR spectra were recorded using DMSO-*d_6_* as solvent and tetramethylsilane as internal standard using a Bruker AVANCE 400 spectrometer operating at 400 MHz and 100 MHz, respectively (the spectrometer was manufactured Bruker Corporation, Billerica, MA, USA, USA and it is located at the Department of Chemistry-Universidad del Valle). The mass spectra were scanned on a Shimadzu GCMS-QP 2010 time-of-flight (TOF) spectrometer (equipped with a direct inlet probe), operating at 70 eV. The mass spectrometer is located at the Chemistry Department-Universidad del Valle, Cali, Colombia, and it was manufactured by Shimadzu Corporation, Kyoto, Japan.

Experimental procedure for the preparation of pyrazolo[3,4-b]pyridines **MM-3a–i** and 2-(3,3-dimethylindolin-2-ylidene)-3-((3-methyl-1-phenyl-1H-pyrazol-5-yl)imino)propanal (intermediate **6**) is presented in the Supplementary Material.

### 4.2. Computational Simulation

The molecules studied here (A1899, PK-THPP, DR16, DR16.1, BAY1000493, BAY2341237, **MM-3a–i**, and **6**) were sketched and processed using LigPrep with the force field OPLS-2005 [42]; possible states of ionization at pH 7.0 ± 2.0 were generated with Epik [43]. Then, the designed compounds were screened against the 3-point pharmacophore of TASK-3 blockers reported by our group [9] by using the software Phase [44]. Up to 50 conformers per molecule were generated during the search, and the other Phase screening settings were maintained by default.

For docking simulations, we used the human TASK-3 model reported previously [45] and the ligands A1899, PK-THPP, **MM-3a–i**, and intermediate **6**. The TASK-3 homology model was aligned to the human TASK-1 crystallographic structure (PDB code: 6VR3) and the grid box was centered using the atomic coordinates of the co-crystallized ligand BAY 1,000,493 [10], keeping the residues from the active PK-THPP site in TASK-3 inside the grid box: T93, L122, Q126, G231, G236, A237, L239, L244, L247, and T248 [40]. The docking simulation was performed using the software Glide [46] with the standard precision (SP) scoring function, obtaining 10 poses per docking simulation.

We computed the physicochemical descriptors, ADME (absorption, distribution, metabolism, and excretion), pharmacokinetic properties, and druglike nature of the studied compounds (A1899, PK-THPP, **MM-3a–i**, and intermediate **6**) by using the SwissADME server [38]. Briefly, 42 descriptors were predicted, including physicochemical, lipophilicity, water solubility, and pharmacokinetics properties. From these descriptors, SwissADME assessed the compounds’ acceptability based on a bioavailability score (drug likeness) [38].

### 4.3. Optical and Electrophysiological Characterization of New TASK-3 Blockers

The ligands **MM-3b**, **MM-3f**, and **MM-3h**, as well as the reported blocker PK-THPP (as a positive control), were screened by FLIPR–FMP [39]. This technique detects ion channels modulation by increasing or decreasing the fluorescent signal as cellular membrane potential changes [39,47]. CHO–K1 cells, stably expressing *h*TASK-3 channels, were loaded with the membrane potential sensitive fluorescent dye and incubated at 37 °C to ensure dye distribution across the cell membrane. All compounds were dissolved in DMSO with a final concentration of 0.25% DMSO. We did not use compound concentrations beyond 50 µM in the fluorescence assay to avoid cross-reaction with the voltage sensitive dye. After recording the baseline fluorescence signal (for 10 s), compounds were added, and the fluorescence signal was detected for 120 s. Subsequently the external potassium concentration was increased (from 2 to 40 mM), and the fluorescence signal was detected for another 120 s. The effect of hits ligands was determined indirectly. Inhibition of TASK-3 led to intracellular retention of potassium ions and influx of the fluorescent dye, resulting in increased fluorescence signal.

All two-electrode voltage clamp (TEVC) measurements were performed as previously described [34]. Both compounds (PK-THPP and **MM-3b**) were dissolved in DMSO, aliquoted, stored at −20 °C or room temperature, and added to the external solution just before the voltage clamp recordings. Final DMSO concentration did not exceed 0.001%. Recordings were performed at room temperature after 48 h of *h*TASK3 cRNA injection with a Digidata 1200 series (Axon Instruments, Union City, CA, USA) as an analog to digital (A/D) converter and a TurboTEC 10CD (npi) amplifier. Borosilicate glass capillaries GB 150TF-8P (Science Products, Hofheim, Germany) were pulled with a DMZ-Universal Puller (Zeitz, Martinsried, Germany). Recording glass capillaries had a resistance of 0.5–1.5 MΩ when filled with 3 M KCl solution, whereas ND96 was used as extracellular recording solution, containing NaCl 96 mM, KCl 2 mM, CaCl_2_ 1.8 M, MgCl_2_ 1 mM, and HEPES 5 mM (pH 7.5). With the following protocol, inhibition by TASK-3 blockers was analyzed: a test pulse to 0 mV of 1 s duration from a holding potential of −80 mV, followed by a voltage step to −80 mV for 1 s, and directly followed by another 1 s test pulse to +40 mV. The sweep time interval was 10 s. For IC_50_ measurements, the blocker concentrations used were in the range of 50 nM to 100 µM. Three to seven oocytes were used for each experiment.

## 5. Conclusions

We have first discovered that pyrazolo[3,4-*b*]pyridines are a new emerging family of TASK-3 channel blockers. Here, we reported a novel blocker interacting with *h*TASK-3 (Figure 5), which exhibits low µM activity. Nevertheless, to determine how this compound interacts with TASK-3, site-directed mutagenesis, X-ray crystallography, or Cryo-electron microscopy experiments are required to characterize the molecular determinants governing the interactions between **MM-3b** and *h*TASK-3 channels. Such experiments could identify the key residues involved in **MM-3b**–TASK-3 binding modes and common amino acids of the TASK-3 central cavity identified for other blockers such as PK-THPP. The results presented here allow us to conclude that at least two hydrogen bond acceptors (*A1* and *A2*) and one aromatic ring (*R*) located above the plane *A1* and *A2* (100% satisfied by compound **MM-3b**) are likely required for a proper rational design of any TASK-3 antagonist.

The systematic pipeline implemented by our groups in the past years—including pharmacophore identification, drug design, and experimental validation supported by computational simulations—is useful to identify novel TASK-3 blockers. The implemented pipeline allows us to identify active compounds, and due to the fact that we are starting from a common pharmacophore of active compounds in the nM to µM range, we expected our workflow to produce active compounds in the same range of inhibitory activity. In the future, we will synthesize congeneric series of **MM-3b** as a building block. Hit-to-lead optimization through medicinal chemistry campaigns might lead to obtaining a more potent TASK-3 inhibitor.

## Figures and Tables

**Table 1:** 5-(indol-2-yl)pyrazolo[3,4-*b*]pyridines derivatives **MM-3a–i** and intermediate **6**.

		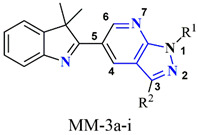						
Product	R^1^	R^2^	Time (min)			Yield * (%)		
			Method			Method		
			A ^a^	B ^b^	C ^c^	A ^a^	B ^b^	C ^c^
**MM-3a** [31]	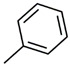	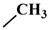	180	15	10	50	40	75
**MM-3b**	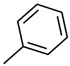	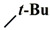	300	20	15	57	33	76
**MM-3c** [31]	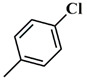	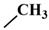	180	20	12	60	72	80
**MM-3d**	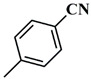	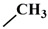	540	35	20	30	72	76
**MM-3e** [31]	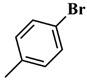	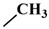	300	25	10	50	41	80
**MM-3f**	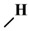	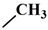	180	16	10	50	67	72
**MM-3g**	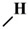	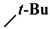	180	20	12	60	54	77
**MM-3h**	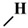	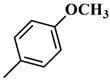	40	16	8	50	73	90
**MM-3i**	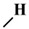	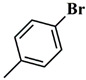	180	15	10	53	62	70
Intermediate **6**	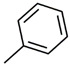	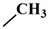	12	12	10	60	75	83

* Isolated yields. ^a^ Method A: AcOH/conventional heating under reflux. ^b^ Method B: AcOH/MWI/110 °C/200 W. ^c^ Method C: AcOH/USI, 20 KHz/500 W.

**Table 2:** Docking of new 5-(indol-2-yl)pyrazolo[3,4-*b*]pyridines **MM-3a–i** and intermediate **6**.

Compound	Docking Score (Kcal/mol)
A1899	−9.478
PK-THPP	−8.961
**MM-3a**	−7.697
**MM-3b**	−8.485
**MM-3c**	−7.802
**MM-3d**	−7.658
**MM-3e**	−7.211
**MM-3f**	−7.409
**MM-3g**	−7.322
**MM-3h**	−8.124
**MM-3i**	−7.969
Intermediate **6**	−7.589

**Table 3:** Physicochemical descriptors calculated with SwissADME [38].

Molecule	MW ^1^	Log P_o/w_ ^2^	Water Solubility (mol/L) ^3^	Pharmacokinetics		
				GI abs ^4^	BBB ^5^	log K_p_ (cm/s) ^6^
A1899	500.54	5.34	1.06 × 10^−6^	High	No	−5.66
PK-THPP	468.59	4.04	4.18 × 10^−6^	High	Yes	−6.1
**MM-3a**	352.43	4.58	2.74 × 10^−6^	High	Yes	−4.96
**MM-3b**	394.51	5.42	3.03 × 10^−7^	High	Yes	−4.3
**MM-3c**	386.88	5.11	7.15 × 10^−7^	High	Yes	−4.73
**MM-3d**	377.44	4.35	3.20 × 10^−6^	High	Yes	−5.32
**MM-3e**	431.33	5.18	3.43 × 10^−7^	High	Yes	−4.96
**MM-3f**	276.34	3.26	8.90 × 10^−5^	High	Yes	−5.69
**MM-3g**	318.42	4.13	9.89 × 10^−6^	High	Yes	−5.02
**MM-3h**	368.43	4.27	5.12 × 10^−6^	High	Yes	−5.38
**MM-3i**	417.3	4.9	7.34 × 10^−7^	High	Yes	−5.17
Intermediate **6**	370.45	4.05	3.98 × 10^−6^	High	Yes	−5.02

^1^ Molecular weight (g/mol); ^2^ Average lipophilicity of iLOGP, XLOGP, WLOGP, MLOGP, and SILICOS-IT predictions; ^3^ SILICOS-IT: Hybrid fragmental/topological method calculated by FILTER-IT program, version 1.0.2, courtesy of SILICOS-IT; ^4^ Gastrointestinal absorption; ^5^ Blood–brain barrier permeation; ^6^ Skin permeation: QSPR model.

## Data Availability

The data presented in this study are available in Supplementary Materials. Raw data is available upon request.

## Supplementary Materials

The following are available online. Figure S1: Comparison of **MM-3f**, **MM-3h**, and PK-THPP at different concentrations interacting with human TASK-3 channels. Figures S2–S22: NMR data collected. Figure S23: Proposed binding site of MM-3b in TASK-3 compared with BAY1000493 interacting with TASK-1 (PDB code: 6RV3). Scheme S1: Synthesis of new 5-(indol-2-yl)pyrazolo[3,4-*b*]pyridines **MM-3a–i**. Table S1: Physicochemical descriptors and drug-likeness properties calculated with SwissADME.

## Sample Availability

Samples of the compounds are available upon authors.
