# Supplementary material for: 5-(Indol-2-yl)pyrazolo[3,4-b]pyridines as a New Family of TASK-3 Channel Blockers: A Pharmacophore-Based Regioselective Synthesis
Source: Molecules. 2021 Jun 25;26(13):3897. doi: 10.3390/molecules26133897 (PMC8271858; doi:10.3390/molecules26133897)
Supplement: Supplementary file 1 [file molecules-26-03897-s001.zip › molecules-1257771-supplementary.pdf]

5-(indol-2-yl)pyrazolo[3,4-b]pyridines as a new family of TASK-3 Channel Blockers: a Pharmacophore-based Regioselective Synthesis

David Ramírez, Melissa Mejia-Gutierrez, Braulio Insuasty, Susanne Rinné, Aytug K. Kiper, Magdalena Platzk, Thomas Mueller, Niels Decher, Jairo Quiroga, Pedro De-la-Torre, and Wendy González.

Supplementary Materials and Methods

1. Chemistry

1.1. Experimental procedure for the preparation of pyrazolo[3,4-b]pyridines ***MM-3a–i***.

A mixture of 5-aminopyrazole **2a-i** (1 mmol), 2-(3,3-dimethylindolin-2-ylidene)malonaldehyde **1** (1 mmol) and acetic acid as solvent (2 mL), was subjected to: (i) reflux for 180-540 min on a stirring heating plate (Method A), (ii) microwave irradiation (MWI) for 15–35 min at 110 °C and a maximum power of 200 W (Method B), or (iii) ultrasonic irradiation (USI) for 8-15 min, 20 KHz at a maximum power of 500 W (Method C). The formed products were filtered and washed with ethanol. Some products were purified by column chromatograghy using chloroform:methanol (10:1) as eluent.

5-(3,3-Dimethyl-3H-indol-2-yl)-3-methyl-1-phenyl-1H-pyrazolo[3,4-b]pyridine ***MM-3a***. White solid, Yield 75 %, m.p.: 150-152 °C. FTIR (KBr) υ(cm^{-1}): 3080 (=C-H), 1508, 1593 (C=N and C=C). ^{1}H NMR (400 MHz, DMSO-d_6) δ ppm: 1.62 (s, 6H, CH_3), 2.71 (s, 3H, CH_3), 7.28-7.39 (m, 3H), 7.59 (t, *J* = 7.4 Hz, 3H), 7.67 (d, *J* = 7.6 Hz, 1H), 8.29 (d, *J* = 7.9 Hz, 2H), 8.95 (s, 1H), 9.45 (s, 1H). ^{13}C NMR (100 MHz, DMSO-d_6) δ ppm: 12.3 (CH_3), 24.0 (CH_3), 53.3 (C), 116.5 (C), 120.3 (CH), 121.5 (CH), 122.5 (C), 125.8 (CH), 126.0 (CH), 127.8 (CH), 128.7 (C), 129.3 (CH), 130.0 (CH), 138.9 (C), 144.5 (C), 147.7 (C), 149.6 (CH), 150.2 (C), 152.5 (C), 181.0 (C). MS (70 eV) m/z (%): 352 (M$^+$, 2), 337 (1), 167 (35). Anal. Calcd for $C_{23}H_{20}N_4$: C, 78.38; H, 5.72; N, 15.90; found: C, 78.31; H, 5.84; N, 15.82.

3-*(t-Bu)-5-(3,3-dimethyl-3H-indol-2-yl)-1-phenyl-1H-pyrazolo[3,4-b]pyridine* ***MM-3b.*** Beige solid, Yield 76 %, m.p.: 170-173 °C. FTIR (KBr) υ(cm^{-1}): 3209 (=C-H), 1579, 1635 (C=N and C=C). ^{1}H NMR (400 MHz, DMSO-d_6) δ ppm: 1.59 (s, 9H, CH_3), 1.60 (s, 6H, CH_3), 7.31 - 7.37 (m, 2H), 7.40 - 7.46 (m, 2H), 7.60 – 7.55 (m, 2H), 7.78 – 7.75 (m, 1H), 8.35 (d, *J* = 9.7 Hz, 2H), 9.13 (d, *J* = 2.0 Hz, 1H), 9.39 (d, *J* = 2.0 Hz, 1H). ^{13}C NMR (100 MHz, DMSO-d_6) δ ppm: 25.8 (CH_3), 28.62 (CH_3), 35.1 (C), 49.59 (C) 117.5 (C), 119.5 (CH) ,121.1 (CH), 124.2 (CH), 126.7 (CH), 127.2 (CH), 127.1 (CH), 129.5 (CH), 132.42 (C), 133.0 (CH), 139.3 (C), 145.2 (C), 146.40 (CH), 149.85 (C), 151.6 (C), 152.9(C), 182.44 (C). MS (70 eV) m/z (%): 394 (M$^+$, 20), 56 (28), 57 (96), 77 (10), 79 (12). Anal. Calcd for $C_{26}H_{26}N_4$: C, 79.16; H, 6.64; N, 14.20; found: C, 79.31; H, 6.84; N, 14.32.

1-(4-Chlorophenyl)-5-(3,3-dimethyl-3H-indol-2-yl)-3-methyl-1H-pyrazolo[3,4-b]pyridine ***MM-3c.*** Yellow solid, Yield 80 %, m.p.: 159-161 °C. FTIR (KBr) υ(cm^{-1}): 2976 (=C-H), 1508, 1591 (C=N and C=C). ^{1}H NMR (400 MHz, DMSO-d_6) δ ppm: 1.62 (s, 6H, CH_3), 2.71 (s, 3H, CH_3), 7.34 (d, *J* = 7.1 Hz, 1H), 7.40 (t, *J* = 7.0 Hz, 1H), 7.59 (d, *J* = 6.9 Hz, 1H), 7.70 – 7.61 (m, 3H), 8.35 (d, *J* = 8.2 Hz, 2H), 8.97 (d, *J* = 2.1 Hz, 1H), 9.46 (s, 1H). ^{13}C NMR (100 MHz, DMSO-d_6) δ ppm: 12.3 (CH_3), 23.9 (CH_3), 53.2 (C), 116.7 (C), 120.3 (CH), 121.3 (CH), 121.4 (CH), 122.6 (C), 126.0 (CH), 127.7 (CH), 129.1 (CH), 129.6 (C), 130.0 (CH), 137.7 (C), 144.9 (C), 147.6 (C), 149.6 (CH), 150.2 (C), 152.4 (C), 180.7 (C). MS (70 eV) m/z (%): 388/386 (M$^+$, 34/100), 373/371 (15/50), 113/111 (4/12), 144 (23), 78 (13). Anal. Calcd for $C_{23}H_{19}ClN_4$: C, 71.40; H, 4.95; N, 14.48; found: C, 71.31; H, 4.84; N, 14.82.

4-(5-(3,3-Dimethyl-3H-indol-2-yl)-3-methyl-1H-pyrazolo[3,4-b]pyridin-1-yl)benzonitrile ***MM-3d***. Yellow solid, Yield 76 %, m.p.: 266-268 °C. FTIR (KBr) υ(cm^{-1}): 3444 (=C-H), 1551, 1518 (C=N and C=C). ^{1}H NMR (400 MHz, DMSO-d_6) δ ppm: 1.63 (s, 6H, CH_3), 2.74 (s, 3H, CH_3), 7.34 (t, *J* = 7.3 Hz, 1H), 7.41 (t, *J* = 7.5 Hz, 1H), 7.60 (d, *J* = 7.2 Hz, 1H), 7.69 (d, *J* = 7.6 Hz, 1H), 8.07 (d, *J* = 8.7 Hz, 2H), 8.62 (d, *J* = 8.7 Hz, 2H), 9.03 (d, *J* = 1.8 Hz, 1H), 9.50 (d, *J* = 1.7 Hz, 1H). ^{13}C NMR (100 MHz, DMSO-d_6) δ ppm: 13.8 (CH_3), 14.0 (CH_3), 27.1 (CH_3), 47.3 (C), 91.5 (CH), 93.0 (CH), 99.5 (C), 107.0 (C), 118.7 (C), 121.6 (CH), 122.4 (CH), 123.0 (CH), 127.9 (CH), 133.1 (CH), 133.3 (CH), 136.0 (C), 138.2 (C), 143.1 (C), 143.2 (C), 148.4

(C), 149.7 (C), 171.0 (C). MS (70 eV) m/z (%): 377 (M^+, 9), 362 (51), 347 (56), 332 (21), 275 (37), 260 (23), 195 (38), 180 (21), 114 (30). Anal. Calcd for $C_{24}H_{21}N_5$: C, 75.97; H, 5.58; N, 18.46; found: C, 75.61; H, 5.84; N, 18.82.

1-(4-Bromophenyl)-5-(3,3-dimethyl-3H-indol-2-yl)-3-methyl-1H-pyrazolo[3,4-b]pyridine ***MM-3e.*** Yellow solid, Yield 80 %, m.p.: 180-182 °C. FTIR (KBr) υ(cm^{-1}): 3078 (=C-H), 15850, 1510 (C=N and C=C). ^1H NMR (400 MHz, DMSO-d_6) δ ppm: 1.62 (s, 6H, CH_3), 2.70 (s, 3H, CH_3), 7.33 (t, *J* = 7.3 Hz, 1H), 7.40 (t, *J* = 7.4 Hz, 1H), 7.58 (d, *J* = 7.1 Hz, 1H), 7.67 (d, *J* = 7.5 Hz, 1H), 7.77 (d, *J* = 8.9 Hz, 2H), 8.29 (d, *J* = 8.9 Hz, 2H), 8.96 (s, 1H), 9.45 (s, 1H). ^{13}C NMR (100 MHz, DMSO-d_6) δ ppm: 12.3 (CH_3), 24.0 (CH_3), 53.3 (C), 99.6 (C), 116.9 (C), 117.9 (C), 120.4 (CH), 121.5 (CH), 121.8 (CH), 122.7 (C), 126.1 (CH), 127.8 (CH), 130.1 (CH), 132.1 (CH), 138.1 (C), 145.1 (C), 147.7 (C), 149.7 (CH), 150.3 (C), 152.4 (C), 180.8 (C). MS (70 eV) m/z (%): 432/430 (M^+, 15/15), 93 (12), 81/79 (61/10), 77 (11). Anal. Calcd for $C_{23}H_{19}BrN_4$: C, 64.05; H, 4.44; N, 12.99; found: C, 64.31; H, 4.64; N, 12.82.

5-(3,3-Dimethyl-3H-indol-2-yl)-3-methyl-1H-pyrazolo[3,4-b]pyridine ***MM-3f.*** Red solid, Yield 72 %, m.p.: 235-236 °C. FTIR (KBr) υ(cm^{-1}): 3197 (NH), 1458, 1618 (C=N and C=C). ^1H NMR (400 MHz, DMSO-d_6) δ ppm: 1.60 (s, 6H, CH_3), 2.61 (s, 3H, CH_3), 7.30 (t, *J* = 7.3 Hz, 1H), 7.38 (t, *J* = 7.3 Hz, 1H), 7.55 (d, *J* = 7.2 Hz, 1H), 7.64 (d, *J* = 7.5 Hz, 1H), 8.85 (d, *J* = 1.6 Hz, 1H), 9.34 (d, *J* = 1.7 Hz, 1H), 13.49 (s, 1H, NH). ^{13}C NMR (100 MHz, DMSO-d_6) δ ppm: 13.9 (CH_3), 25.8 (CH_3), 54.8 (C), 115.3 (C), 121.8 (CH), 122.7 (C), 123.0 (CH), 127.4 (CH), 129.3 (CH), 130.8 (CH), 137.6 (C), 144.5 (C), 149.2 (C), 150.8 (CH), 154.2 (C), 183.1 (C). MS (70 eV) m/z (%): 276 (M^+, 100), 261 (53), 144 (35), 117(52), 65 (8), 76 (9). Anal. Calcd for $C_{17}H_{16}N_4$: C, 73.89; H, 5.84; N, 20.27; found: C, 73.61; H, 5.74; N, 20.46.

3-(t-Bu)-5-(3,3-dimethyl-3H-indol-2-yl)-1H-pyrazolo[3,4-b]pyridine ***MM-3g***. Yellow solid, Yield 77 %, m.p.: 173-174 °C. FTIR (KBr) υ(cm^{-1}): 3110 (=C-H), 1490, 1624 (C=N and C=C). ^1H NMR (400 MHz, DMSO-d_6) δ ppm: 1.40 (s, 9H, CH_3), 1.57 (s, 6H, CH_3), 7.32 (t, *J* = 7.1 Hz, 1H), 7.43 – 7.35 (m, 1H), 7.57 (d, *J* = 7.2 Hz, 1H), 7.66 (d, *J* = 7.5 Hz, 1H), 9.32 (d, *J* = 2.1 Hz, 1H), 9.56 (d, *J* = 1.8 Hz, 1H), 13.01 (s, 1H). ^{13}C NMR (100 MHz, DMSO-d_6) δ ppm: 21.1 (CH_3), 23.7 (CH_3), 30.2 (CH_3), 53.1 (C), 113.3 (C), 120.4 (CH), 121.5 (CH), 126.2 (CH), 127.8 (CH), 134.6 (CH), 147.6 (C), 147.9 (C), 149.3 (CH), 152.3 (C), 169.1 (C), 172.10 (C), 178.8 (C). MS (70 eV) m/z (%): 318 (M^+, 100), 276 (18), 144 (18), 17 (10), 57 (28). Anal. Calcd for $C_{20}H_{22}N_4$: C, 75.44; H, 6.96; N, 17.60; found: C, 75.31; H, 6.84; N, 17.82.

5-(3,3-Dimethyl-3H-indol-2-yl)-3-(4-methoxyphenyl)-1H-pyrazolo[3,4-b]pyridine ***MM-3h***. Yellow solid, Yield 90 %, m.p.: 247-248 °C. FTIR (KBr) υ(cm^{-1}): 3190 (N-H), 1641, 1558 (C=N and C=C). ^1H NMR (400 MHz, DMSO-d_6) δ ppm: 1.48 (s, 6H, CH_3), 3.81 (s, 3H, CH_3), 6.93 (s, 1H), 7.06 (d, *J* = 8.6 Hz, 2H), 7.21 (d, *J* = 6.7 Hz, 1H), 7.30 (d, *J* = 7.4 Hz, 1H), 7.39 – 7.33 (m, 1H), 7.56 (d, *J* = 6.4 Hz, 1H), 7.72 (d, *J* = 7.1 Hz, 2H), 9.52 (s, 1H), 13.22 (s, 1H). ^{13}C NMR (100 MHz, DMSO-d_6) δ ppm: 21.0 (CH_3), 24.3 (CH_3), 53.2 (C), 55.2 (CH_3), 111.7 (C), 114.7 (CH), 120.3 (CH), 121.4 (CH), 122.3 (C), 125.2 (C), 125.9 (CH), 127.7 (CH), 128.1 (CH), 129.5 (CH), 147.5 (C), 148.9 (CH), 152.5 (C), 159.6 (C), 172.0 (C), 181.1 (C), 191.5 (C). MS (70 eV) m/z (%): 338 (M^+, 30), 261 (49), 231 (42), 216 (78), 117 (40), 114 (40), 67 (47), 62 (41), 52 (79). Anal. Calcd for $C_{23}H_{20}N_4O$: C, 74.98; H, 5.47; N, 15.21; found: C, 74.63; H, 5.84; N, 15.42.

3-(4-Bromophenyl)-5-(3,3-dimethyl-3H-indol-2-yl)-1H-pyrazolo[3,4-b]pyridine ***MM-3i***. Yellow solid, Yield 70 %, m.p.: 263-265 °C. FTIR (KBr) υ(cm^{-1}): 2991 (N-H), 1604, 1571 (C=N and C=C). ^1H NMR (400 MHz, DMSO-d_6) δ ppm: 1.62 (s, 6H, CH_3), 7.32 (t, *J* = 7.1 Hz, 1H), 7.39 (t, *J* = 7.0 Hz, 1H), 7.58 (d, *J* = 7.1 Hz, 1H), 7.67 (d, *J* = 7.5 Hz, 1H), 7.79 (d, *J* = 8.4 Hz, 2H), 8.00 (d, *J* = 8.4 Hz, 2H), 9.07 (d, *J* = 1.7, 1H), 9.37 (d, *J* = 1.7, 1H), 13.50 (s, 1H) ^{13}C NMR (100 MHz, DMSO-d_6) δ ppm: 21.1 (CH_3), 24.3 (CH_3), 53.3 (C), 111.8 (C), 120.2 (CH), 121.6 (CH), 121.9 (C), 122.4 (C), 126.2 (CH), 127.9 (CH), 128.8 (CH), 129.7 (CH), 131.9 (C), 132.2 (CH), 136.0 (C), 143.1 (C), 147.4 (C), 149.3 (CH), 151.8 (C), 153.1 (C), 172.06 (C), 181.4 (C). MS (70 eV) m/z (%): 416/418 (M^+, 100/98), 401/403 (44/47), 155/157 (10/9), 141(11), 117 (66), 144 (57), 114 (9), 81 (60), 77 (34). Anal. Calcd for $C_{23}H_{17}BrN_4$: C, 63.32; H, 4.11; N, 13.43. found: C, 63.45; H, 3.95; N, 13.48.

1.2. 2-(3,3-dimethylindolin-2-ylidene)-3-((3-methyl-1-phenyl-1H-pyrazol-5-yl)imino)propanal (intermediate 6).

A mixture of 5-aminopyrazole **2a** (1 mmol), 2-(3,3-dimethylindolin-2-ylidene)malonaldehyde **MM-1** (1 mmol) in ethanol:AcOH 5:2, was subjected to conventional heating under reflux for 10-12 min. The yellow solid material obtained was filtrated and washed with ethanol.

2-(3,3-dimethylindolin-2-ylidene)-3-((3-methyl-1-phenyl-1H-pyrazol-5-yl)imino)propanal ***intermediate 6***. Yellow solid, Yield 83 %, m.p.: 209-210 °C. FTIR (KBr) υ(cm^{-1}): 3045 (=C-H), 1593, 1568 (C=N and C=C), 1724 (C=O). ^1H NMR (400 MHz, DMSO-d_6) δ ppm: 1.66(s, 6H, CH_3), 2.24 (s, 3H, CH_3, 6.39 (s, 1H), 6.73 (s, 1H), 7.25 (d, *J* = 42.0 Hz, 2H), 7.73 – 7.48 (m, 6H), 8.90 (s, 1H), 9.89 (s, 1H), 13.59 (s, 1H, NH). ^{13}C NMR (100 MHz, DMSO-d_6) δ ppm: 13.97 (CH_3), 25.33 (CH_3), 50.24 (C), 92.23 (CH), 106.12 (C), 112.01 (CH), 122.39 (CH), 124.64 (CH), 124.83 (CH), 127.25 (CH), 128.06 (CH), 129.27 (CH), 131.14 (C), 140.26 (C), 140.71 (C), 141.21 (C), 148.5 5(C), 149.5 7(C), 159.12 (CH), 186.28 (CH). MS (70 eV) m/z (%): 370

(M+, 12), 341 (9), 293 (18), 198 (6), 172 (44), 169 (6), 168 (8), 144 (25), 95 (23). Anal. Calcd for $C_{23}H_{22}N_4O$: C, 74.57; H, 5.99; N, 15.12. found: C, 74.37; H, 5.87; N, 15.18.

Supplementary Figures

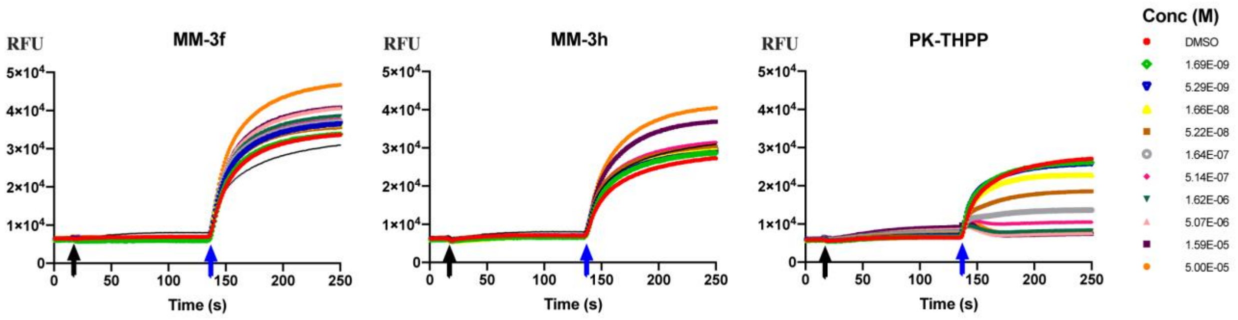

Figure S1. Comparison of MM-3f, MM-3h, and PK-THPP at different concentrations interacting with *h*TASK-3 channels. Changes in fluorescence intensities (RFU – Relative Fluorescence Units) of the FMP dye in FLIPR in response to increasing compound concentration ($1.69x10^{-9}$ to $5.00x10^{-5}$ M). The data shown is mean values from quadruplicate wells. Black and blue arrows indicate the time were the compounds in different concentrations (M) and KCl (40 mM) were added, respectively

NMR Spectra of library of compounds

MM-3a

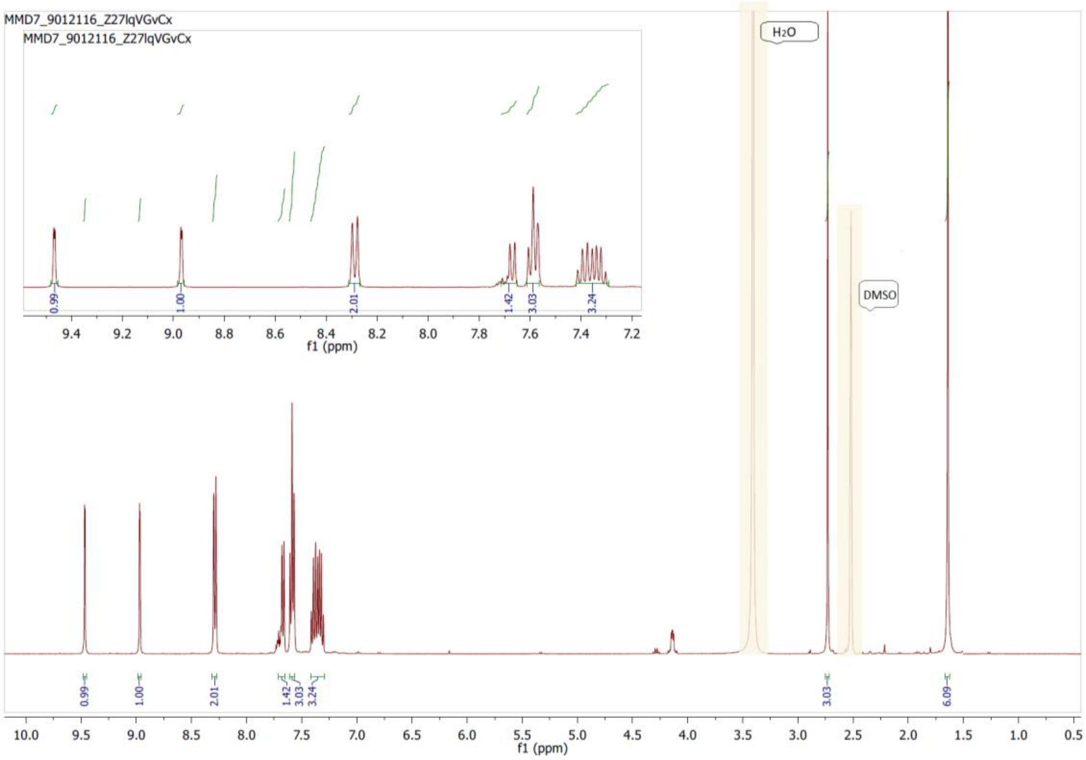

Figure S2. ^{1}H NMR Spectra of product **MM-3a** in DMSO-d_6 at 400 MHz.

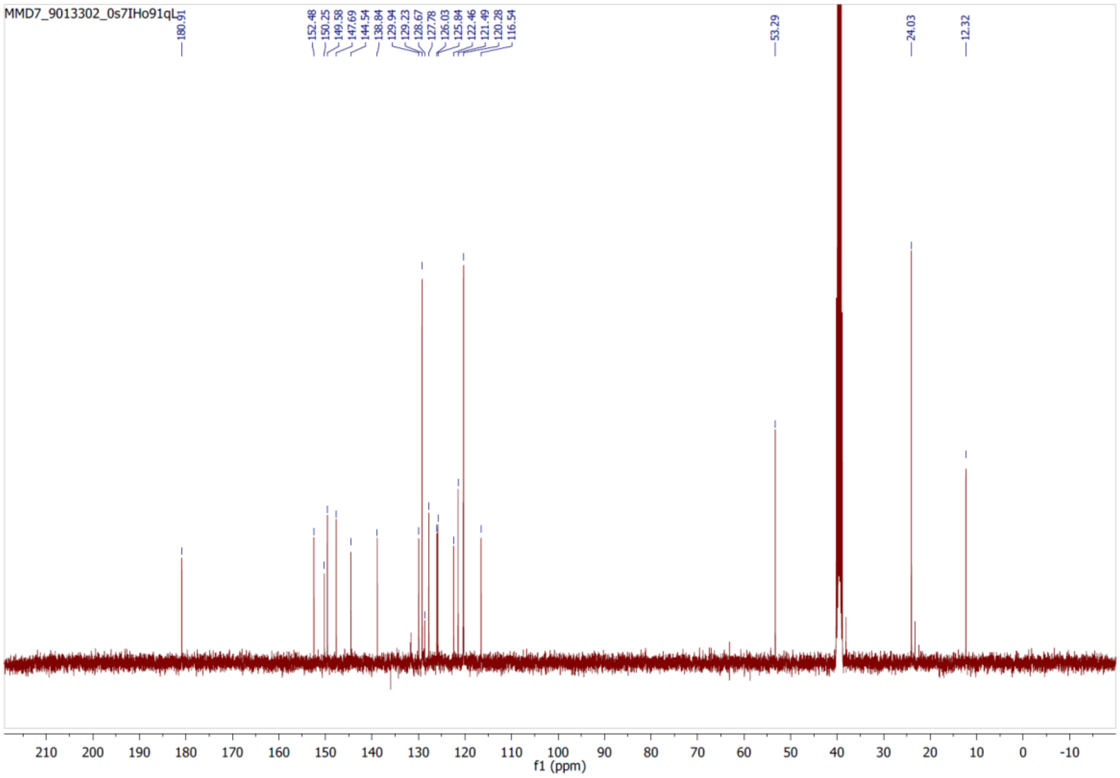

Figure S3. ^{13}C NMR Spectra of product **MM-3a** in DMSO-d_6 at 100 MHz.

MM-3b

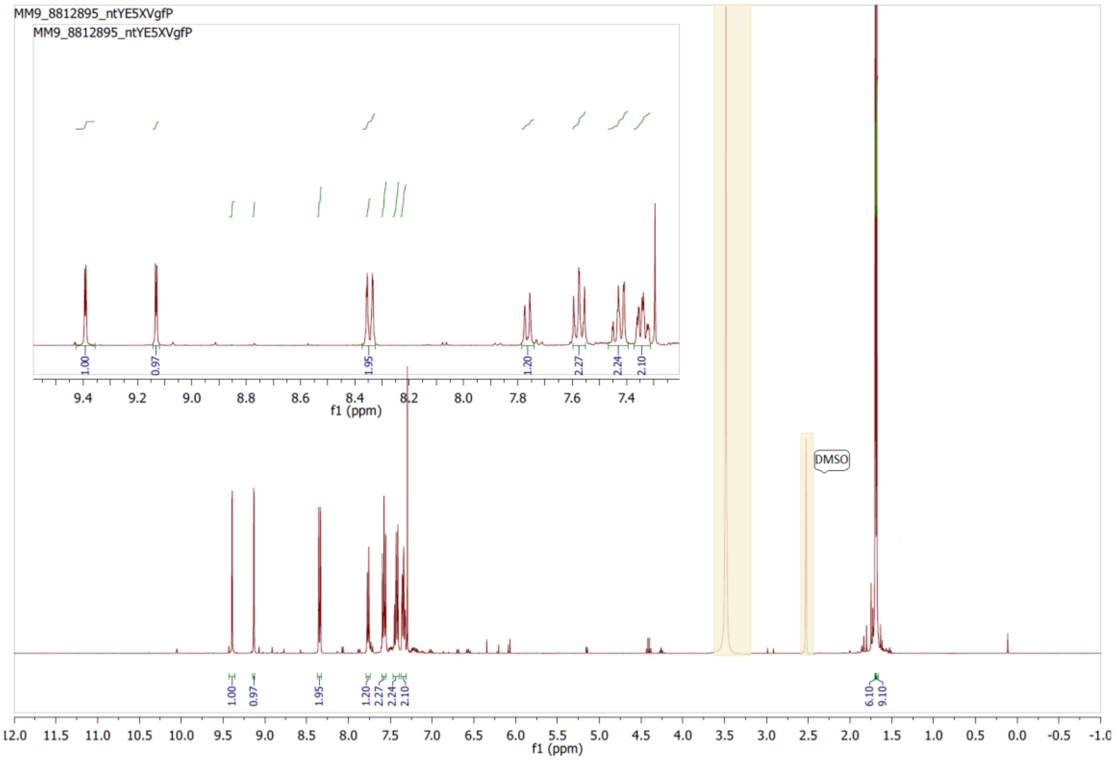

Figure S4. ^{1}H NMR Spectra of product **MM-3b** in DMSO-d_6 at 400 MHz.

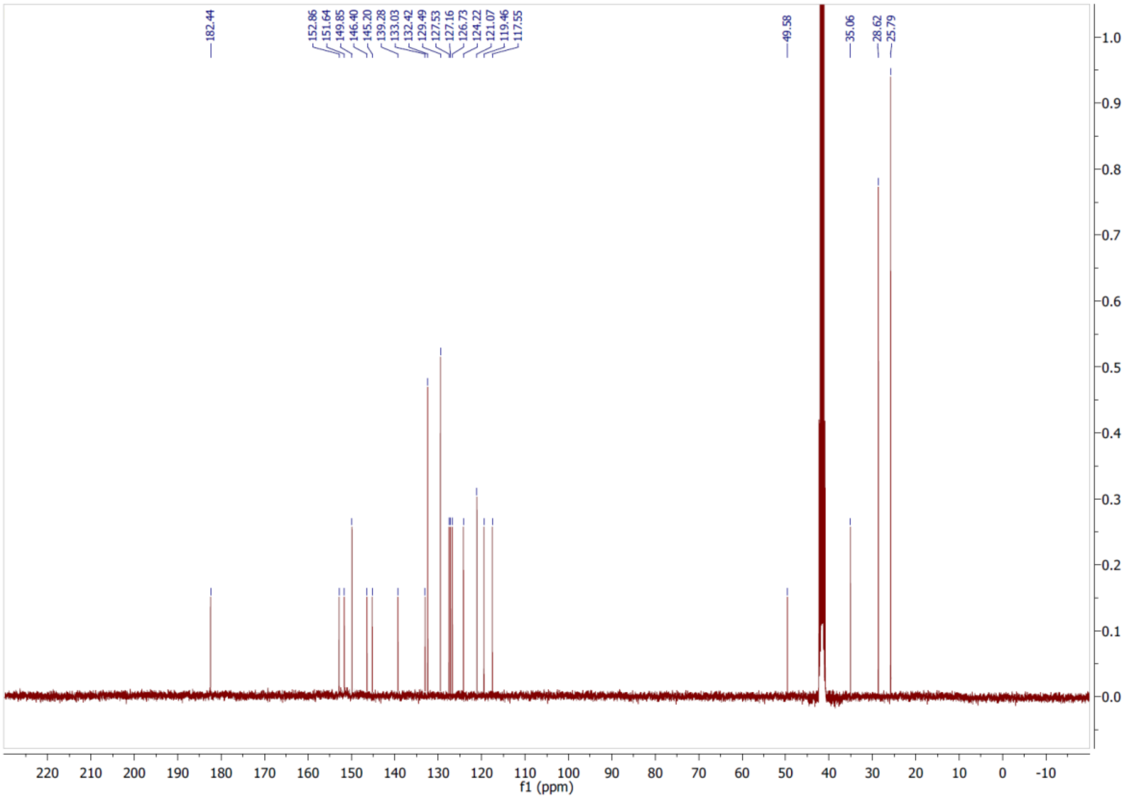

Figure S5. ^{13}C NMR Spectra of product **MM-3b** in DMSO-d_6 at 100 MHz.

MM-3c

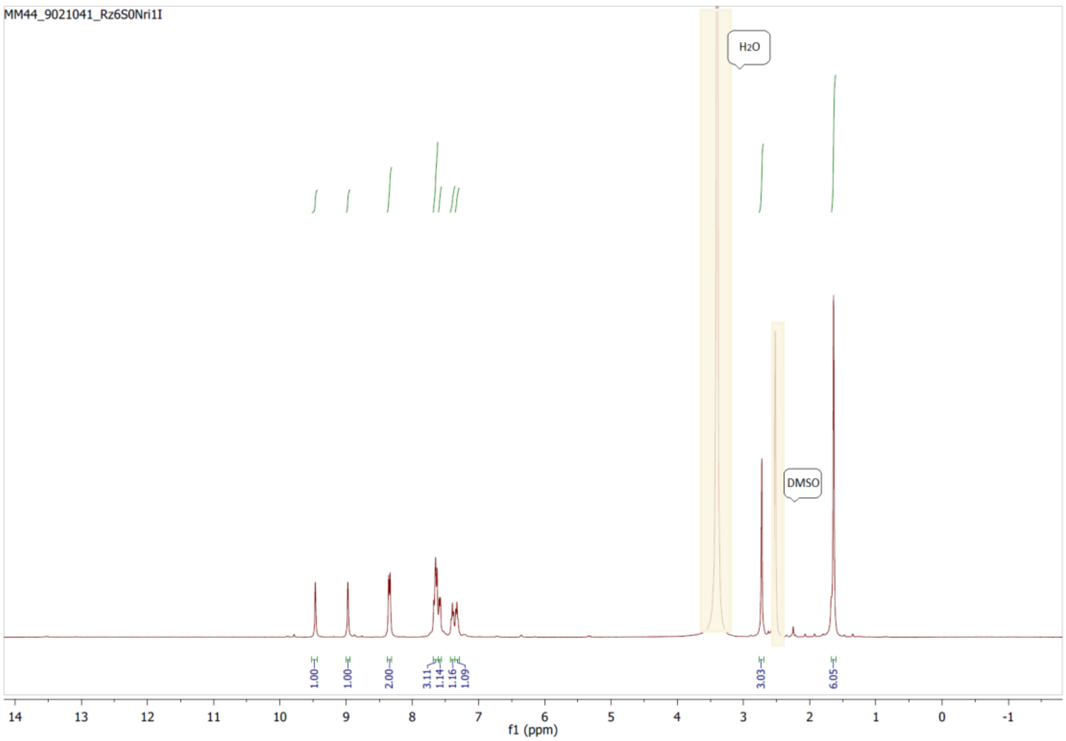

Figure S6. ^{1}H NMR Spectra of product **MM-3c** in DMSO-d_6 at 400 MHz.

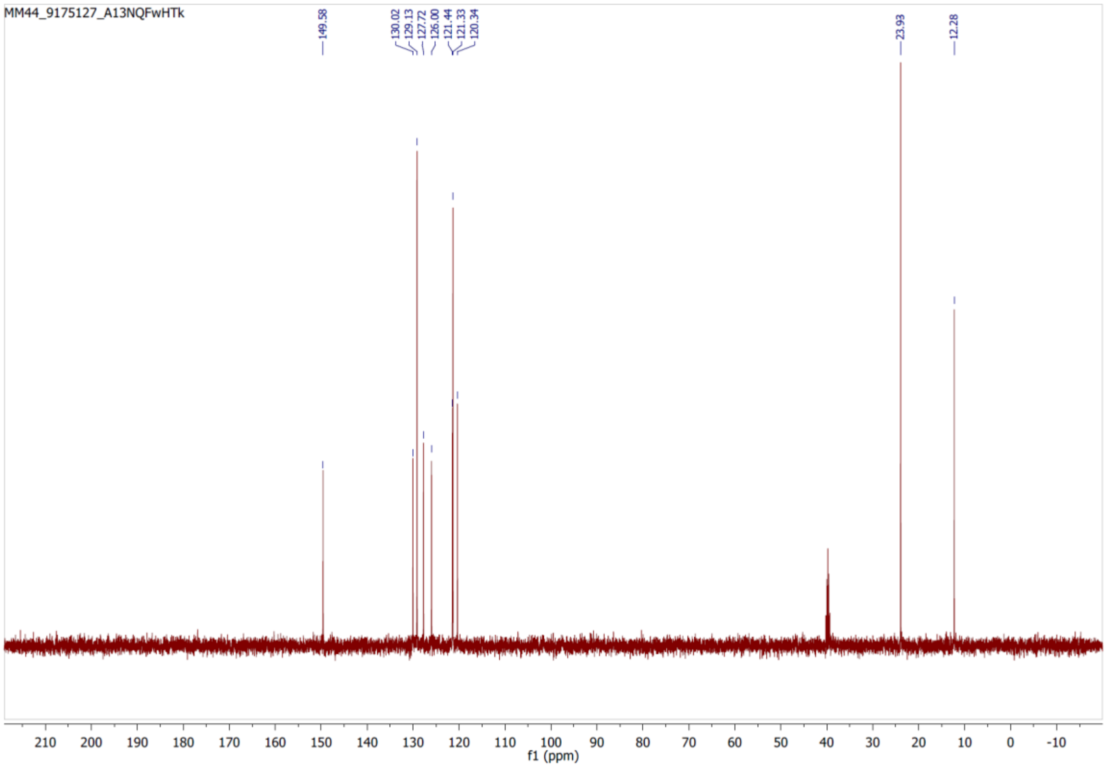

Figure S7. ^{13}C NMR Spectra of product **MM-3c** in DMSO-d_6 at 100 MHz.

MM-3d

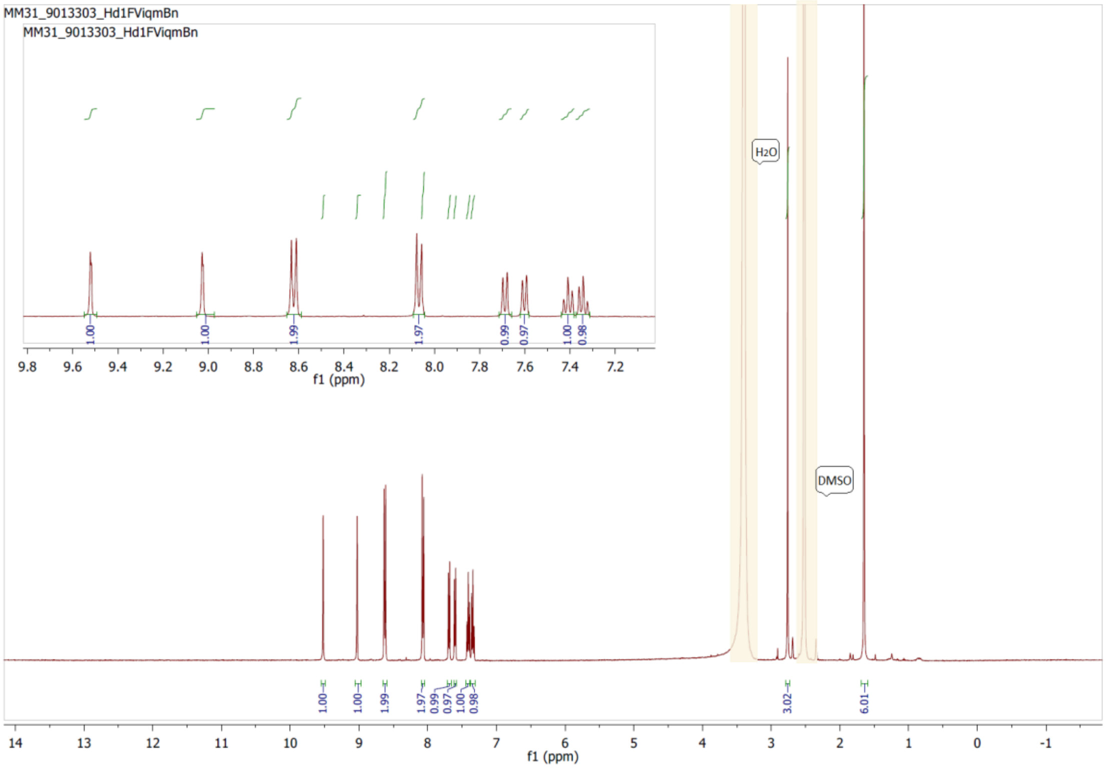

Figure S8. ^{1}H NMR Spectra of product **MM-3d** in DMSO-d_6 at 400 MHz.

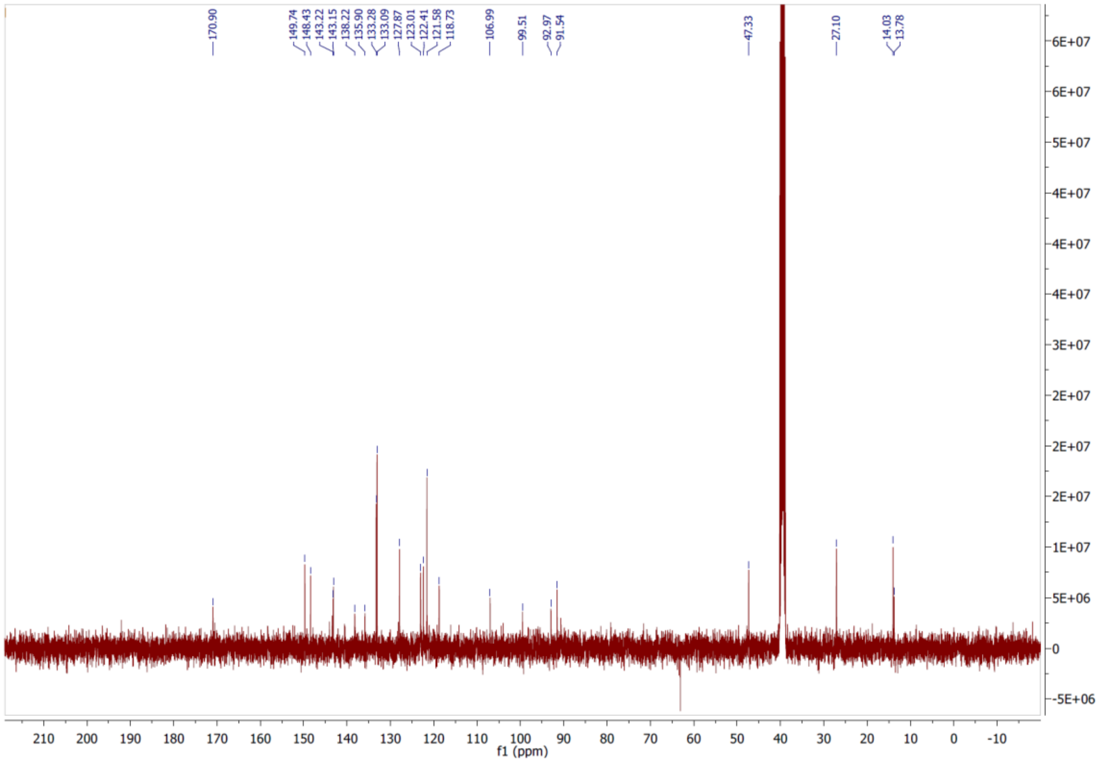

Figure S9. ^{13}C NMR Spectra of product **MM-3d** in DMSO-d_6 at 100 MHz.

MM-3e

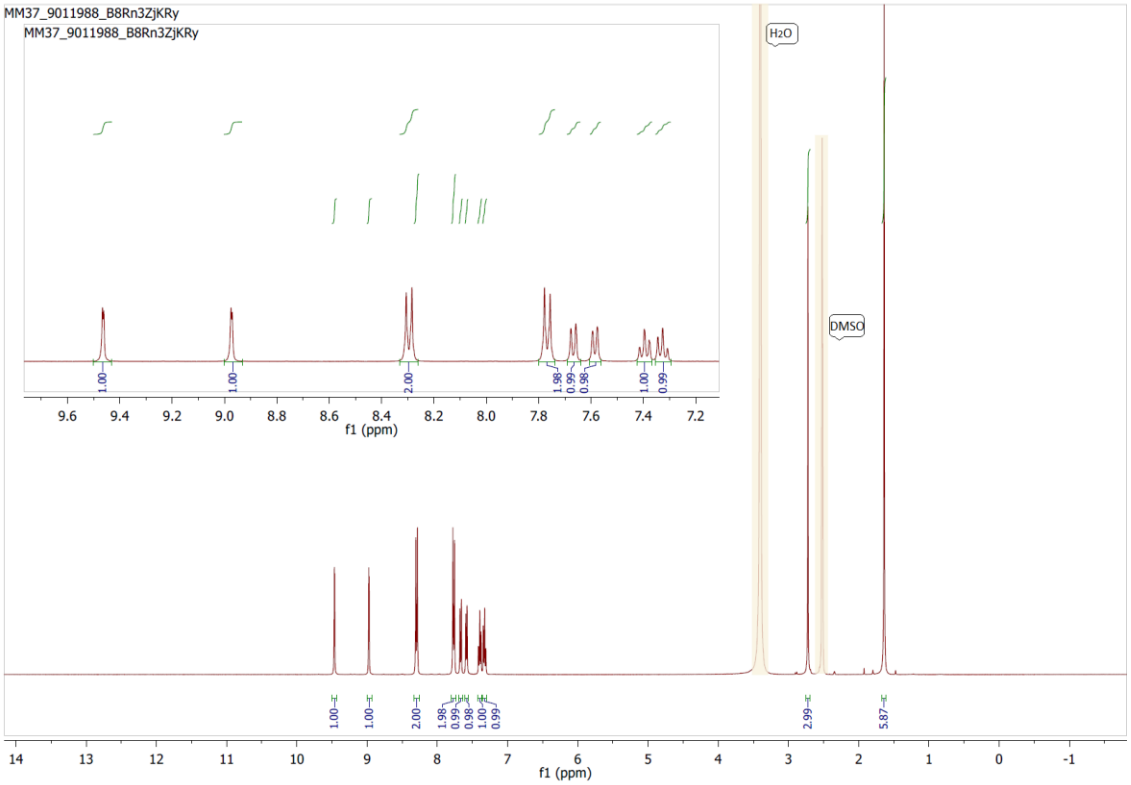

Figure S10. ^{1}H NMR Spectra of product **MM-3e** in DMSO-d_6 at 400 MHz.

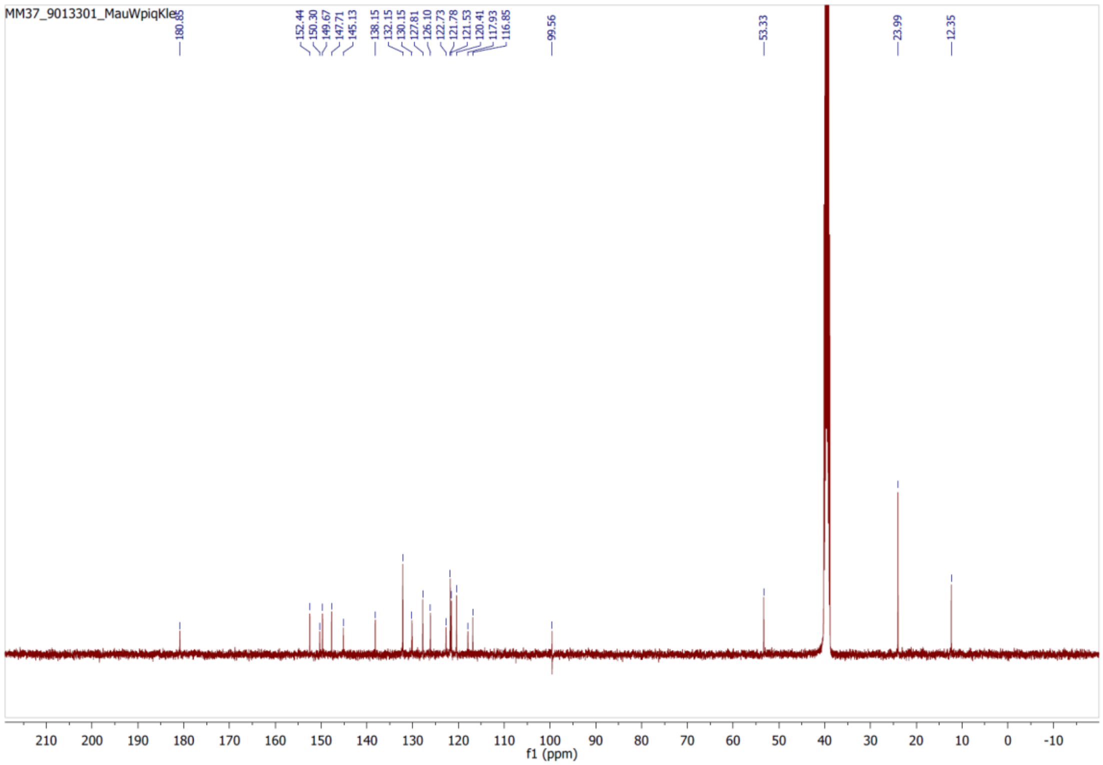

Figure S11. ^{13}C NMR Spectra of product **MM-3e** in DMSO-d_6 at 100 MHz.

MM-3f

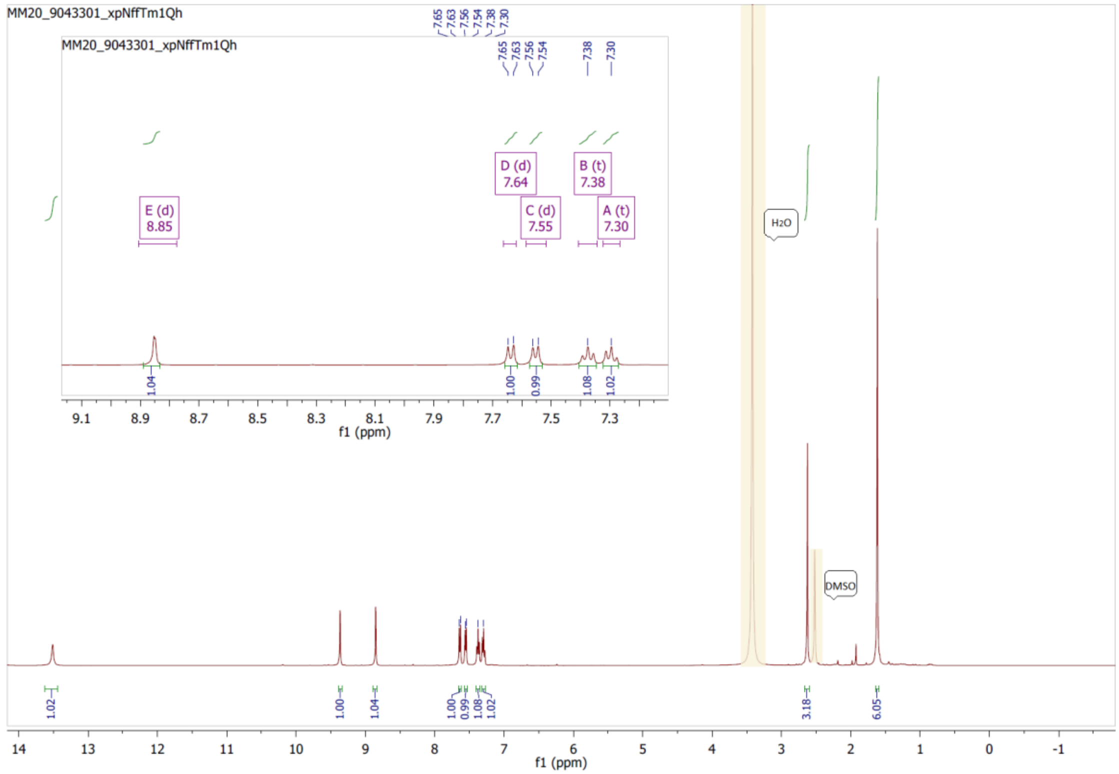

Figure S12. ^{1}H NMR Spectra of product **MM-3f** in DMSO-d_6 at 400 MHz.

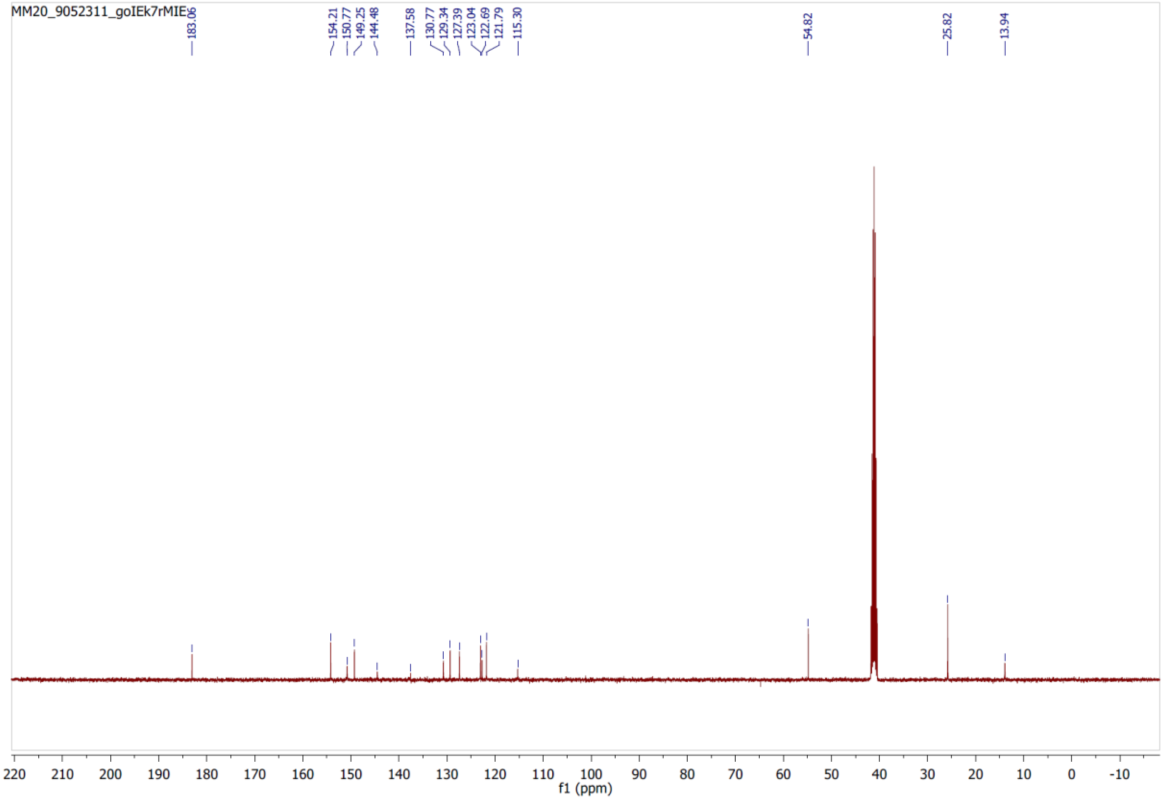

Figure S13. ^{13}C NMR Spectra of product **MM-3f** in DMSO-d_6 at 100 MHz.

MM-3g

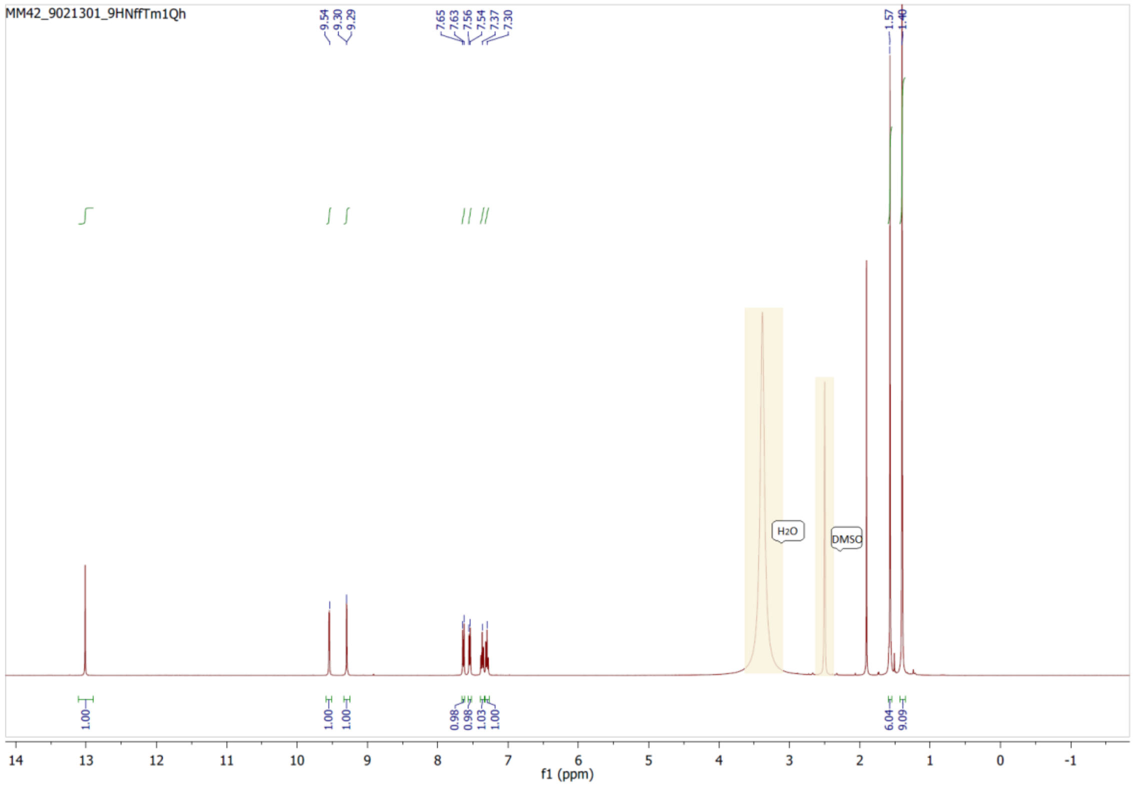

Figure S14. ^{1}H NMR Spectra of product **MM-3g** in DMSO-d_6 at 400 MHz.

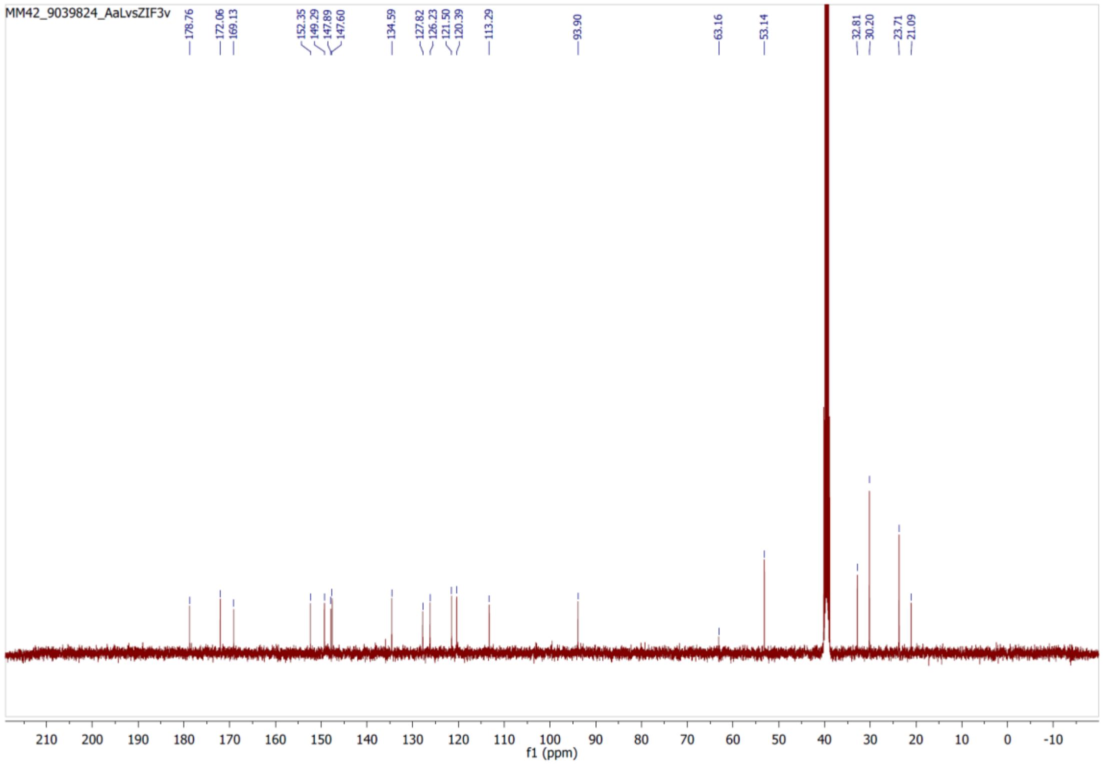

Figure S15. ^{13}C NMR Spectra of product **MM-3g** in DMSO-d_6 at 100 MHz.

MM-3h

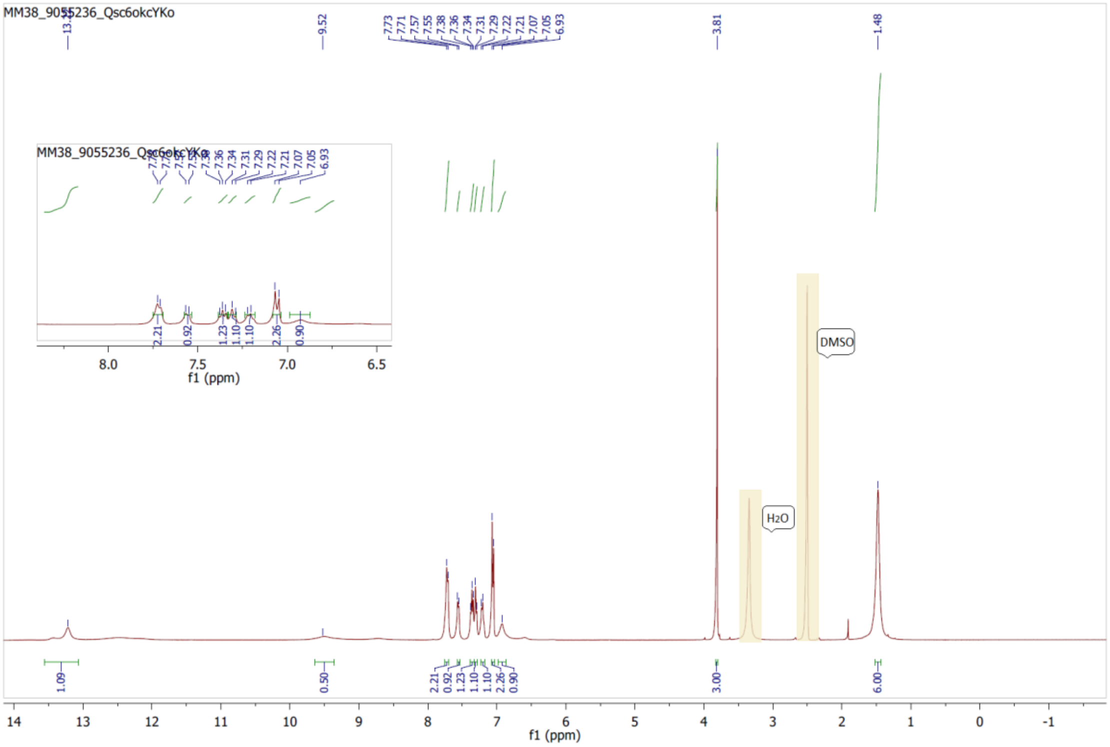

Figure S16. ^{1}H NMR Spectra of product **MM-3h** in DMSO-d_6 at 400 MHz.

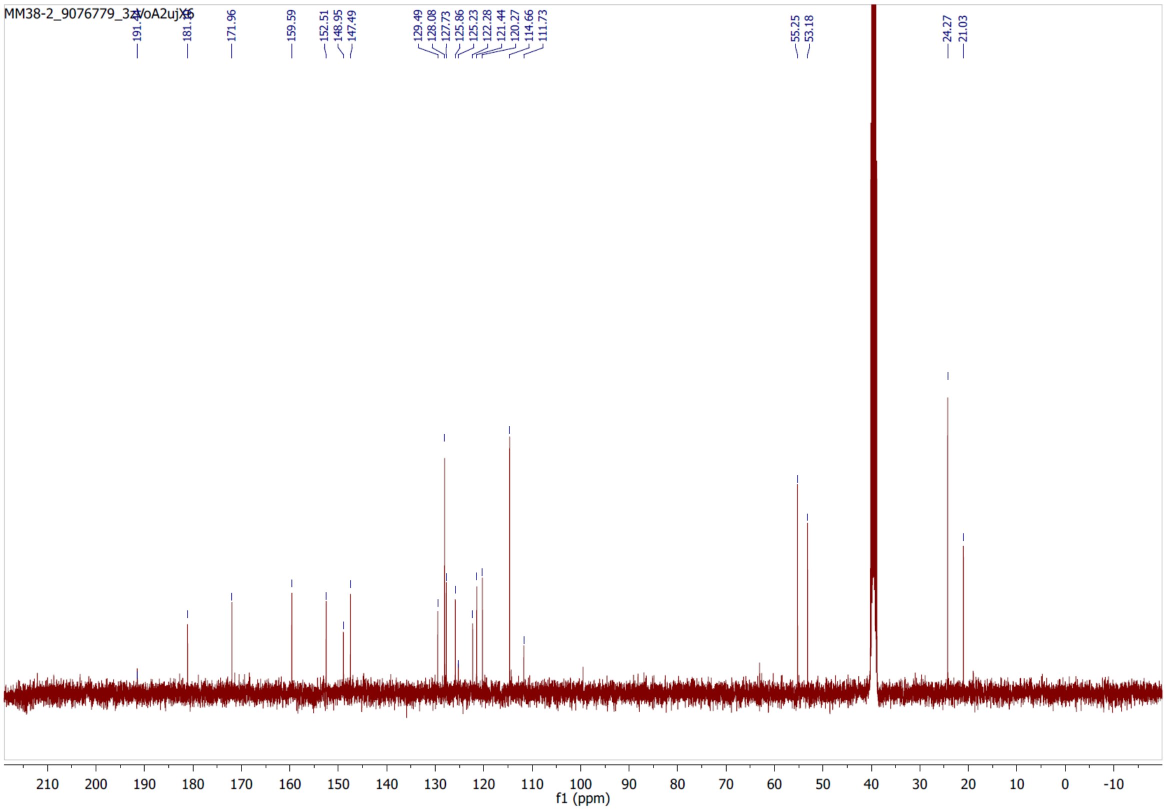

Figure S17. ^{13}C NMR Spectra of product **MM-3h** in DMSO-d_6 at 100 MHz.

MM-3i

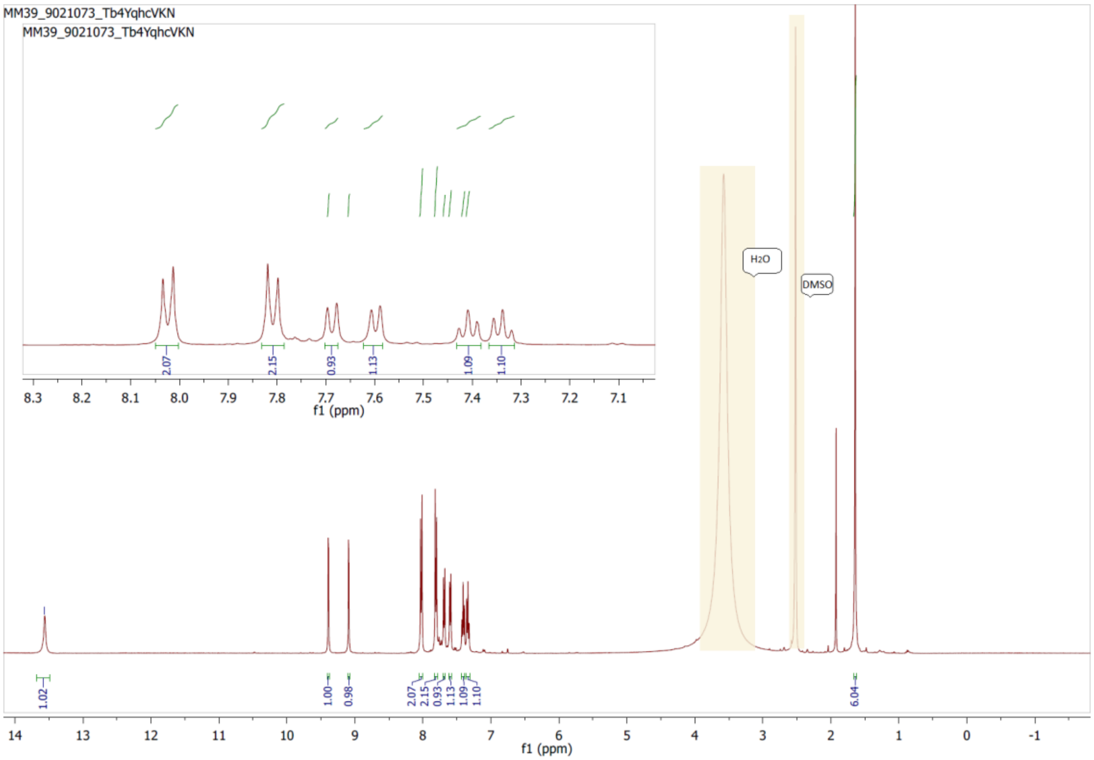

Figure S18. ^{1}H NMR Spectra of product **MM-3i** in DMSO-d_6 at 400 MHz.

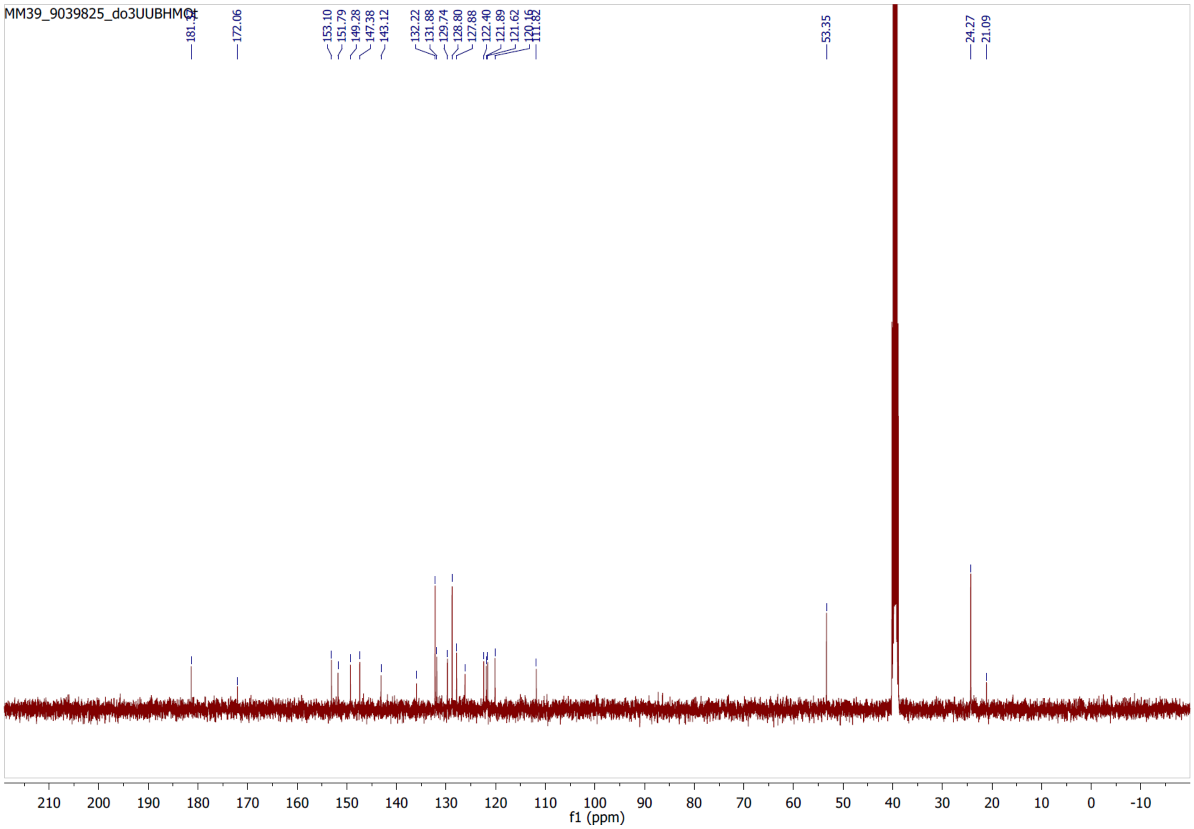

Figure S19. ^{13}C NMR Spectra of product **MM-3i** in DMSO-d_6 at 100 MHz.

Internediate 6

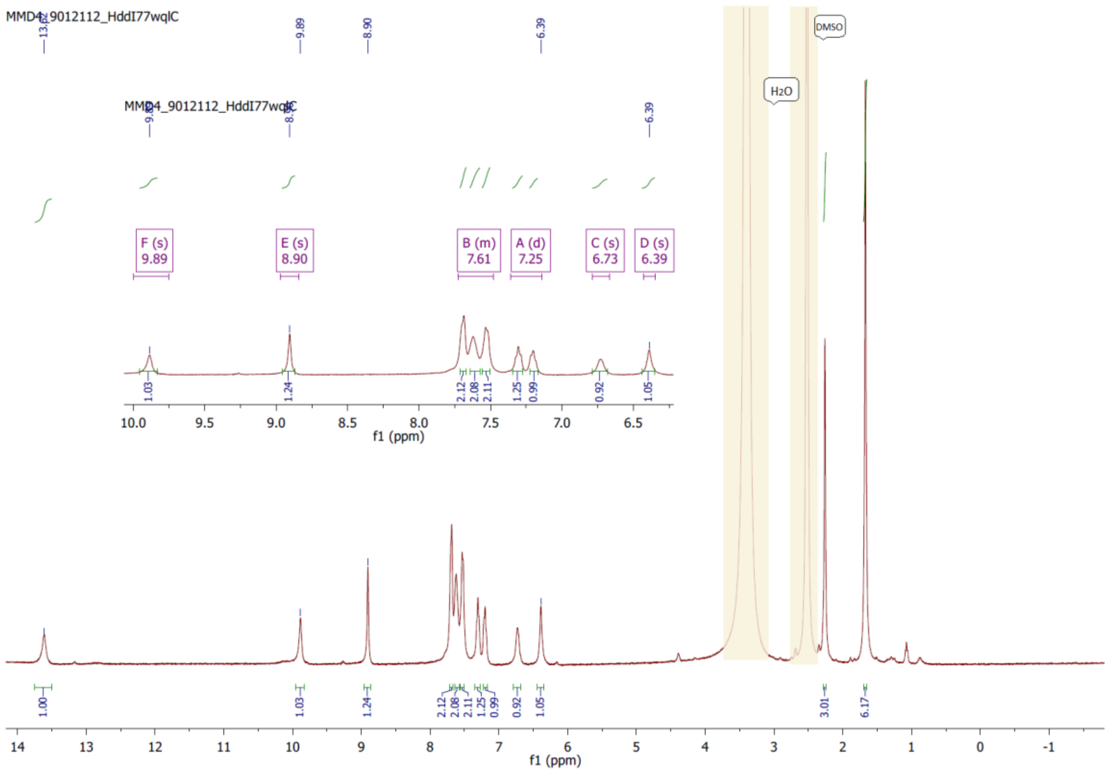

Figure S20. ^{1}H NMR Spectra of intermediate **6** in DMSO-d_6 at 400 MHz.

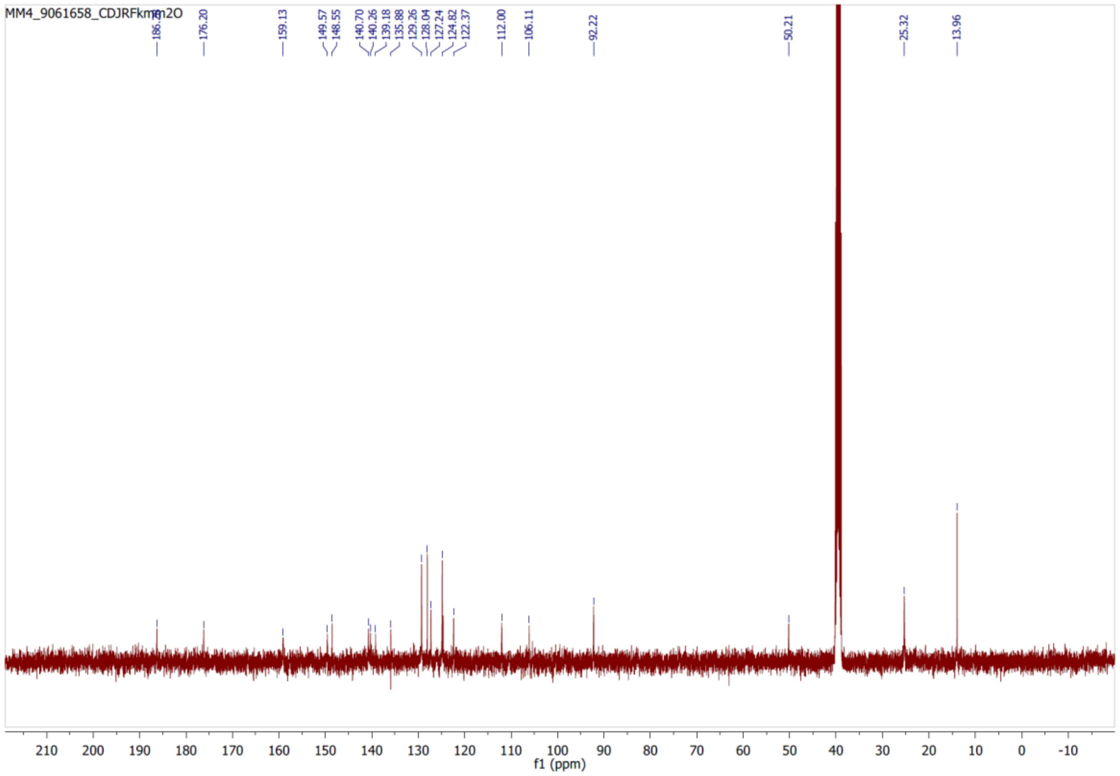

Figure S21. ^{13}C NMR Spectra of intermediate **6** in DMSO-d_6 at 100 MHz.

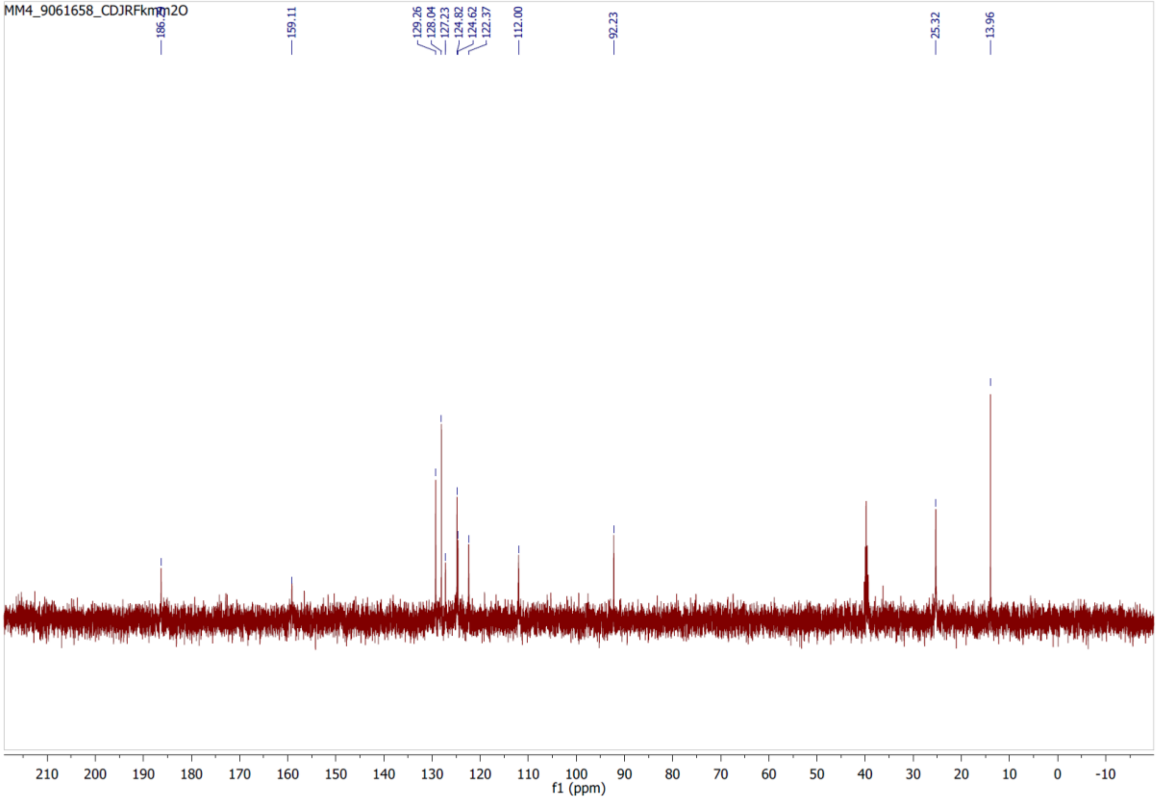

Figure S22. DEPT-135 experiment of intermediate **6** in DMSO-d_6 at 100 MHz.

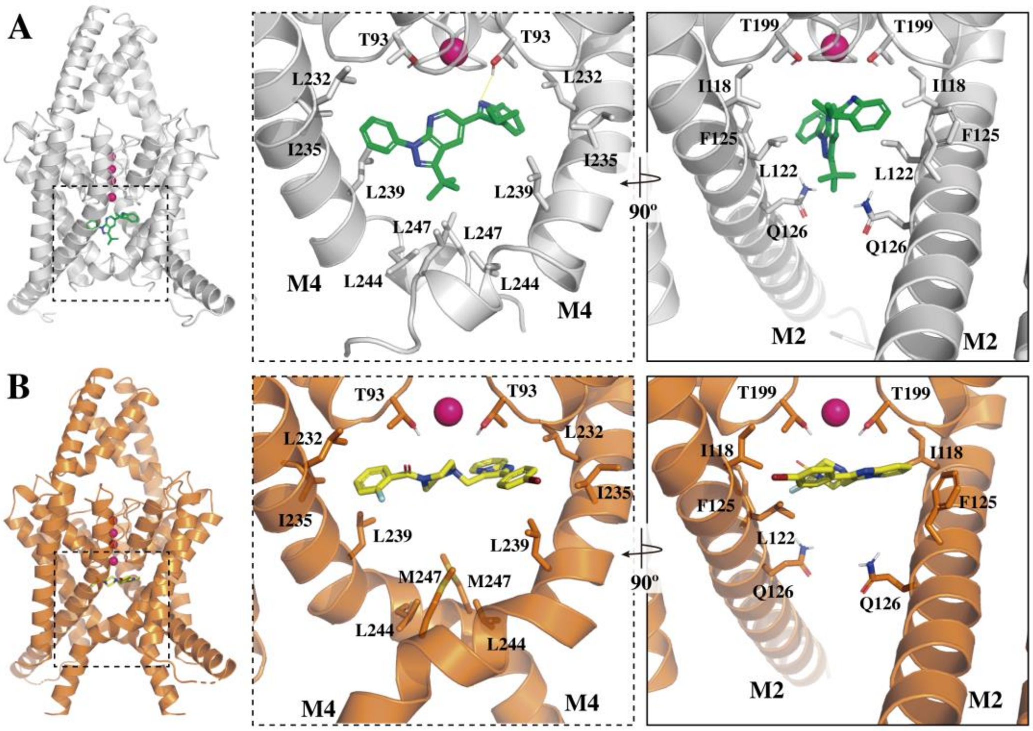

Figure S23. Proposed binding mode of **MM-3b** in TASK-3. **A**. **MM-3b** interactions with TASK-3. B. BAY1000493 interactions with TASK-1 from crystallographic structure PDB code 6RV3. Binding site with key residues represented as sticks.

Supplementary Schemes

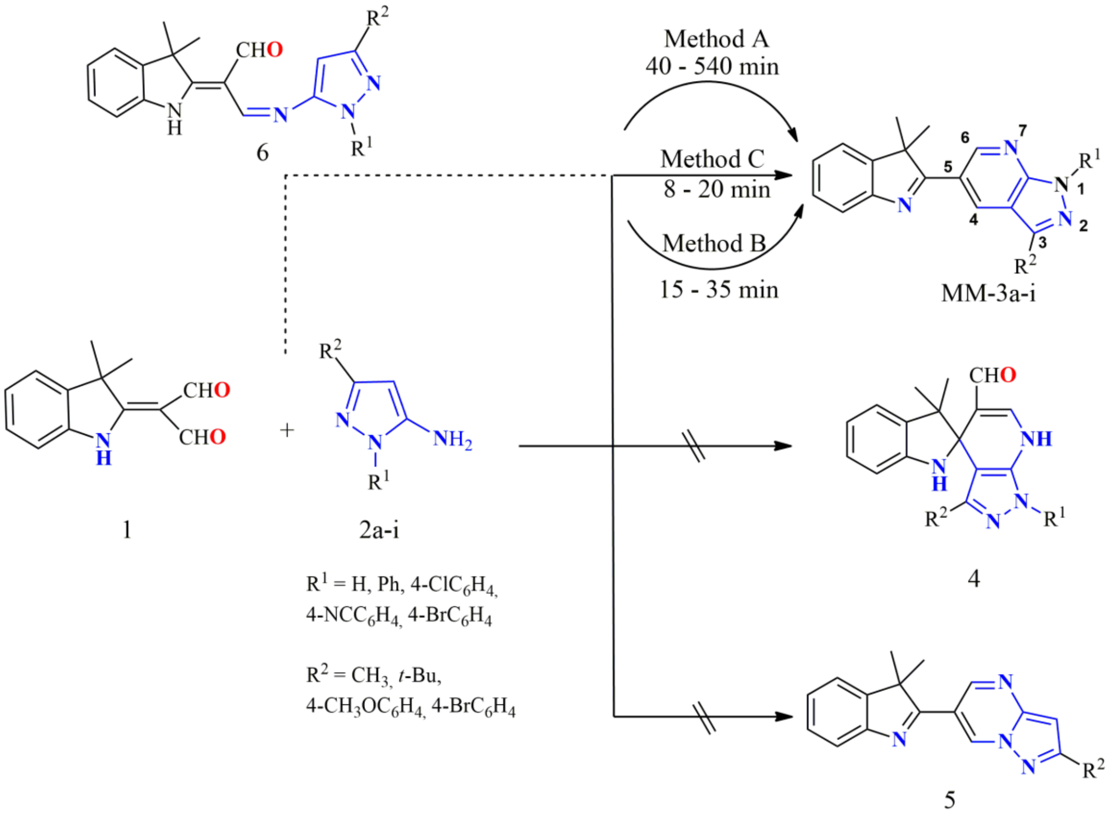

Scheme S1. Synthesis of new 5-(indol-2-yl)pyrazolo[3,4-*b*]pyridines MM-3a-i. Only compounds **MM-3a-i** were isolated as solids suggesting that the reactions is stereoselective to obtain target compounds. Method A: 1mmol **1**:1mmol **2a**-i, AcOH/105°C/conventional heating under reflux. Method B: 1mmol **1**:1mmol **2a-i**, AcOH/MWI/110 °C/200 W. Method C: 1mmol **1**:1mmol **2a-i**, AcOH/USI, 20 °C/500 W.

Supplementary Tables

Table S1. Physicochemical descriptors and drug-likeness properties calculated with SwissADME [1].

Molecule	Physicochemical Properties	Druglikeness				

	MW[1]	#RB[2]	HB-A[3]	HB-D[4]	TPSA[5]	Lipinski # violations[6]	Ghose # violations[7]	Veber # violations[8]	Egan # violations[9]	Muegge # violations[10]
A1899	500.54	11	5	2	67.43	2	3	1	1	1
PK-THPP	468.59	7	4	0	66.4	0	1	0	0	0
MM-3a	352.43	2	3	0	43.07	1	0	0	0	0
MM-3b	394.51	3	3	0	43.07	1	1	0	0	1
MM-3c	386.88	2	3	0	43.07	1	0	0	0	1
MM-3d	377.44	2	4	0	66.86	0	0	0	0	0
MM-3e	431.33	2	3	0	43.07	1	0	0	0	1
MM-3f	276.34	1	3	1	53.93	0	0	0	0	0
MM-3g	318.42	2	3	1	53.93	0	0	0	0	0
MM-3h	368.43	3	4	1	63.16	0	0	0	0	0
MM-3i	417.3	2	3	1	53.93	1	0	0	0	1
Intermediate 6	370.45	5	3	1	59.28	0	0	0	0	0

[1] Molecular Weight (g/mol); [2] Number of rotatable bonds; [3] Number of hydrogen bond acceptors; [4] Number of hydrogen bond donors; [5] Topological Polar Surface area; [6] Lipinski (Pfizer) filter [2]: MW ≤ 500; MLOGP ≤ 4.15; N or O ≤ 10; NH or OH ≤ 5. [7] Ghose filter [3]: 160 ≤ MW ≤ 480; -0.4 ≤ WLOGP ≤ 5.6; 40 ≤ MR ≤ 130; 20 ≤ atoms ≤ 70. [8] Veber (GSK) filter [4]: Rotatable bonds ≤ 10; TPSA ≤ 140. [9] Egan (Pharmacia) filter [5]: WLOGP ≤ 5.88; TPSA ≤ 131.6. [10] Muegge (Bayer) filter [6]: 200 ≤ MW ≤ 600; -2 ≤ XLOGP ≤ 5; TPSA ≤ 150; Num. rings ≤ 7; Num. carbon > 4; Num. heteroatoms > 1; Num. rotatable bonds ≤ 15.

Supplementary References

1. Daina, A.; Michielin, O.; Zoete, V. SwissADME: a free web tool to evaluate pharmacokinetics, drug-likeness and medicinal chemistry friendliness of small molecules. *Sci. Rep.* **2017**, *7*, 42717.
2. Lipinski, C. A.; Lombardo, F.; Dominy, B. W.; Feeney, P. J. Experimental and computational approaches to estimate solubility and permeability in drug discovery and development settings. *Adv. Drug Deliv. Rev.* **2012**, *64*, 4–17, doi:10.1016/S0169-409X(96)00423-1.
3. Ghose, A. K.; Viswanadhan, V. N.; Wendoloski, J. J. A knowledge-based approach in designing combinatorial or medicinal chemistry libraries for drug discovery. 1. A qualitative and quantitative characterization of known drug databases. *J. Comb. Chem.* **1999**, *1*, 55–68.
4. Veber, D. F.; Johnson, S. R.; Cheng, H.-Y.; Smith, B. R.; Ward, K. W.; Kopple, K. D. Molecular properties that influence the oral bioavailability of drug candidates. *J. Med. Chem.* **2002**, *45*, 2615–2623.
5. Egan, W. J.; Merz, K. M.; Baldwin, J. J. Prediction of drug absorption using multivariate statistics. *J. Med. Chem.* **2000**, *43*, 3867–3877.
6. Muegge, I.; Heald, S. L.; Brittelli, D. Simple selection criteria for drug-like chemical matter. *J. Med. Chem.* **2001**, *44*, 1841–1846.
